# Discovery of a novel double-stranded DNA virus associated with ant labial gland disease reveals its long-term interaction with ants

**DOI:** 10.1128/jvi.01178-25

**Published:** 2025-09-17

**Authors:** Shengqiang Jiang, Liangliang Zhang, Xingyu Guo, Jianchao Li, Jing Hu, Hong He, Hongying Chen

**Affiliations:** 1 College of Life Sciences, Northwest A&F University 12469, Yangling, Shaanxi, China; 2 Key Laboratory of National Forestry and Grassland Administration for Control of Forest Biological Disasters in Western China, College of Forestry, Northwest A&F University 12469, , Yangling, Shaanxi, China; 3 Institute of Future Agriculture, Northwest A&F University 12469, Yangling, Shaanxi, China; Wageningen University & Research, Wageningen, Netherlands

**Keywords:** *Camponotus japonicus*, labial gland disease, dsDNA virus, phylogenetic analysis, endogenous viral element

## Abstract

Large double-stranded DNA (dsDNA) viruses have been shown to have a wide host range in insects. However, their infection in ants has not yet been described. In this study, we report the identification of a novel filamentous virus, Camponotus japonicus labial gland disease virus (CjLGDV), from the enlarged labial glands of *Camponotus japonicus*. Transmission electron microscopy revealed numerous non-enveloped nucleocapsids in the nuclei and enveloped envelopment intermediates in the cytoplasm of infected gland cells. Genome sequencing analysis showed that CjLGDV possesses a circular dsDNA genome of 142 kb. Comparative analysis identified a closely related virus from *Anoplolepis gracilipes,* which was named Anoplolepis gracilipes labial gland disease virus (AgLGDV). CjLGDV and AgLGDV have common genomic characteristics and key conserved genes with the viral members in the order *Lefavirales*, class *Naldaviricetes*, as well as additional genes uniquely shared with Apis mellifera filamentous virus (AmFV) and AmFV-like virus (AmFLV). Phylogenetic analysis places CjLGDV and AgLGDV in a distinct lineage within *Naldaviricetes*, probably representing two members of a novel virus family distantly related to AmFV and AmFLV. By mining public databases, LGDV-related endogenous viral elements were identified in the genomes of multiple ant species, suggesting widespread viral gene integration and a deep evolutionary association between these viruses and their ant hosts. Collectively, our findings reveal a previously unrecognized group of ant-infecting viruses that represents a new viral family within the order *Lefavirales*, class *Naldaviricetes*.

**IMPORTANCE:**

Ants, as highly eusocial insects, play vital roles in ecosystems worldwide. While numerous RNA viruses have been documented in ants, no double-stranded DNA (dsDNA) virus has previously been confirmed to infect them. Labial gland disease, reported for decades, lacks a clearly defined cause until now. Here, we identify and characterize a large filamentous dsDNA virus, Camponotus japonicus labial gland disease virus (CjLGDV), from the swollen labial gland of *C. japonicus*, and a closely related Anoplolepis gracilipes labial gland disease virus in *A. gracilipes*. Phylogenetic and genomic analyses of the two viruses support the establishment of a new viral family within the order *Lefavirales*, class *Naldaviricetes*. The discovery of endogenous viral elements related to CjLGDV in multiple ant genomes suggests the historical infection of CjLGDV-like viruses in ants. These findings broaden the known host range of naldaviricetes and shed new light on the diversity, evolution, and host interaction of large dsDNA viruses in arthropods.

## INTRODUCTION

Recent advancements in molecular biology and high-throughput sequencing technologies have substantially enhanced our understanding of virus-host interactions and expanded our knowledge of the global virome (1–4). To date, gene sequences of thousands of species of viruses have been identified in dozens of ant species (5–7). However, the infectivity and transmissibility of these viruses in ants remain largely speculative and unconfirmed. Among those confirmed, the majority are single-stranded RNA viruses belonging to the order *Picornavirales* (7). Some of these viruses, such as Solenopsis invicta virus 3, which alters foraging behavior and increases brood mortality in its host, have been explored as potential biological control agents targeting the invasive red imported fire ant (8–10). Despite extensive research on RNA viruses, documentation of DNA viruses that are infectious for ants is rare. To our best knowledge, the only DNA virus characterized in ants is Solenopsis invicta densovirus (SIDNV), a single-stranded DNA virus found in South American populations of *Solenopsis invicta* (11). Even so, the impact of SIDNV on its host remains unclear. Recent studies on ant viral diversity have uncovered numerous DNA viral genome fragments in several ant species, indicating a broader and more complex landscape of DNA viral infections in ants than previously thought (6).

Endogenous viral elements (EVEs), or viral fossils, are whole or fragmented viral sequences integrated into host genomes after the virus infection and preserved in the host through germline transmission. Recent findings on the distribution of EVEs in ant genomes have significantly advanced our understanding of the viral community composition in ants (12–14). Notably, EVEs related to large double-stranded DNA (dsDNA) viruses have been identified in several ant species, suggesting a historical prevalence of DNA virus infections.

According to the International Committee on Taxonomy of Viruses report, classified members of *Naldaviricetes* are categorized into five families: *Baculoviridae*, *Nudiviridae*, *Hytrosaviridae*, *Filamentoviridae*, and *Nimaviridae* (15, 16). Unclassified filamentous viruses infecting bees, such as Apis mellifera filamentous virus (AmFV) and Apis mellifera filamentous-like virus (AmFLV), share some genetic characteristics with these five virus families. However, phylogenetic analyses suggest that these unclassified filamentous viruses belong to a new viral family (17–19).

A condition referred to as “labial gland disease,” characterized by swollen labial glands and malformed mesosomas, has been reported in more than 10 ant species from Europe, the USA, Japan, and China (20–22). Although the causative agent remains unknown, it has been postulated that the disease may be transmitted by trophallaxis behavior, cannibalism, and probably also vertically from queen to offspring (20, 21). Assumption of a viral pathogen has been proposed to cause the distinct morphological changes, but it has not been confirmed (20, 21).

Recently, we reported the observation of labial gland enlargement symptoms in *Camponotus japonicus*, an ant species widely distributed across East Asia (22). In this study, we identified a novel virus with a circular dsDNA genome from the enlarged labial gland and named it Camponotus japonicus labial gland disease virus (CjLGDV). Morphological characteristics of the virus particles were recorded by transmission electron microscopy. By searching the public database, we also identified a virus closely related to CjLGDV in *Anoplolepis gracilipes*, which we designated as Anoplolepis gracilipes labial gland disease virus (AgLGDV). Phylogenetic analysis was performed to elucidate the evolutionary relationships between LGDVs and other members in *Naldaviricetes*. Furthermore, CjLGDV-related EVEs were characterized in the genomes of various ant species, providing evidence for the long and frequent interaction history between CjLGDV and its ant hosts. These findings expand the known lineage of *Naldaviricetes* and broaden our understanding of their host range.

## RESULTS

### Observation of virus particles in the enlarged labial gland of *Camponotus japonicus*

**Fig 1:**
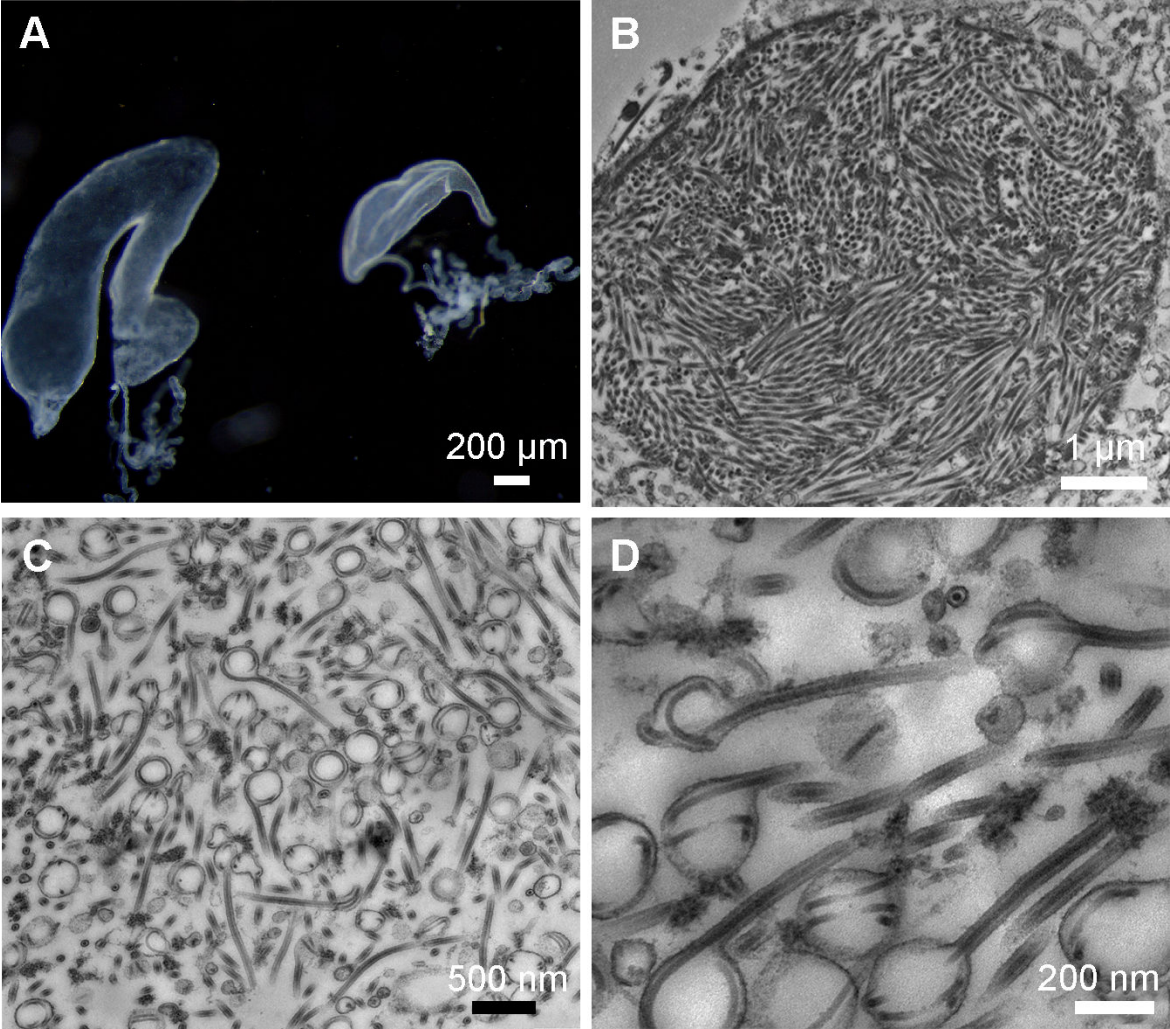
Transmission electron micrographs of Camponotus japonicus labial gland virus in the enlarged labial gland of *C. japonicus*. (**A**) Normal (right) and enlarged labial glands (left) of *C. japonicus*. (**B**) The cell nucleus in an enlarged labial gland cell of *C. japonicus* is full of nucleocapsid-like filamentous particles. (**C**) CjLGDV particles probably in intermediate stages of viral envelopment in the cytoplasm. (**D**) A high magnification image of CjLGDV envelopment intermediates in the cytoplasm.

In our recent report, we found that about one-fifth of the minor workers in a mature nest of *C. japonicus* had swollen labial glands (Fig. 1A) (22). To investigate the cell structure in the enlarged labial gland, the tissue was sliced and examined using transmission electron microscopy. In some enlarged labial gland cells, we observed nuclei stuffed with long filaments, likely viral nucleocapsids in processing (Fig. 1B). In the cytoplasm, numerous virus particles, probably in intermediate stages of viral envelopment, were discovered. Some nucleocapsids appeared coiled within spherical envelopment vesicles around 200 nm, while others were enveloped by a membrane and had filamentous morphology measuring up to 1,000 nm in length and about 65 nm in width. Transitional stages between these two morphologies were also observed (Fig. 1C and D). These observations suggest that the causative agent of the swollen labial gland is probably an enveloped filamentous virus replicated in the nucleus, which is named as Camponotus japonicus labial gland disease virus.

### General features of the viral genome

Based on the observation of numerous nucleocapsid-like filaments in the cell nucleus and the fact that most DNA viruses replicate in the nucleus, we speculated that the causative agent of the labial gland disease could be a DNA virus. To confirm this speculation, DNA was, respectively, extracted from the normal labial gland (LG) and enlarged labial gland (ENLG) of *Camponotus japonicus* and subjected to Illumina sequencing. For the LG group, 91.7% of the reads were mapped to the *Camponotus* genome, while only 48.9% of the reads were mapped to the ant genome in the ENLG group (Fig. S1).

**Fig 2:**
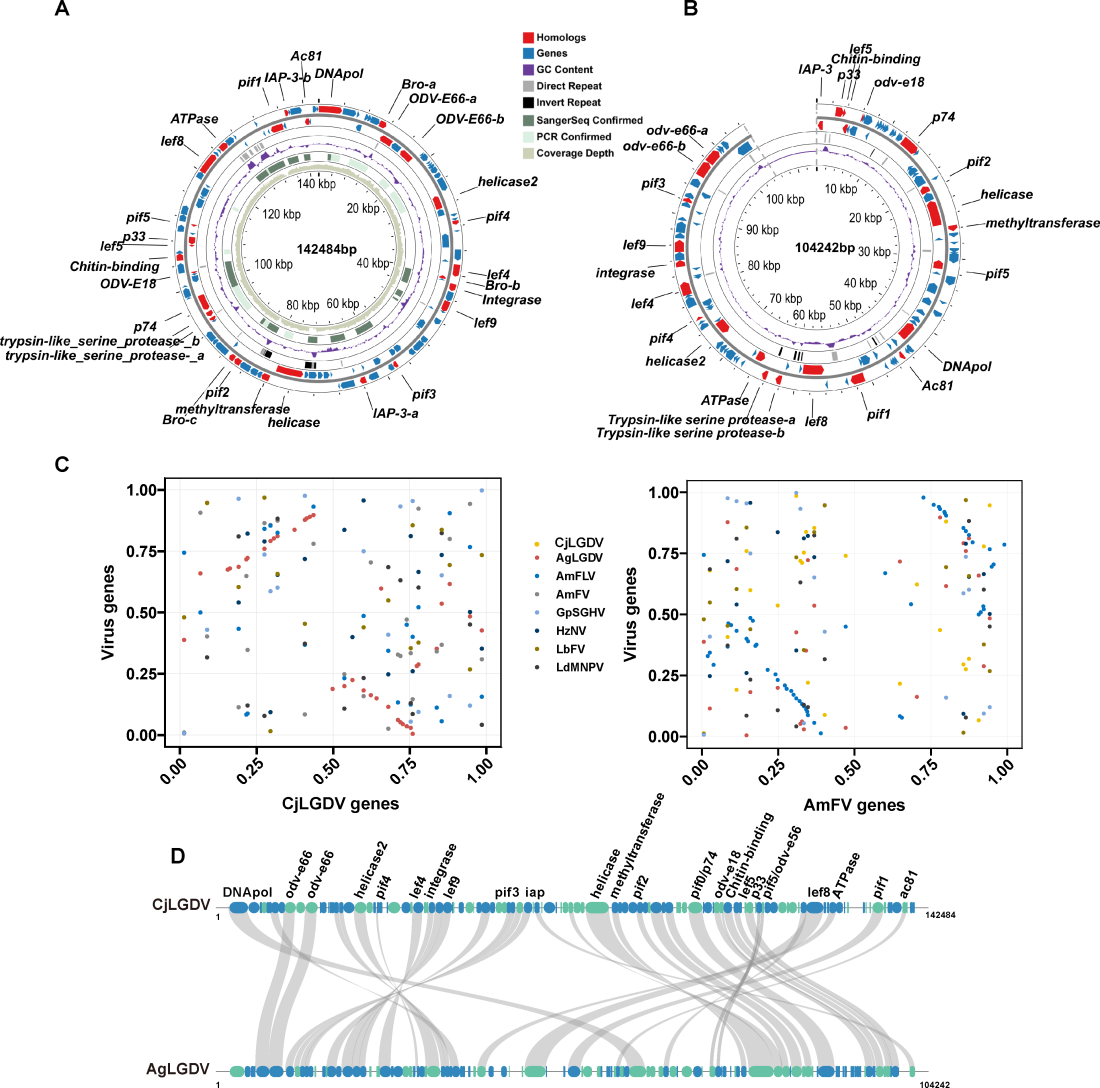
Characterization of CjLGDV and AgLGDV genomes. Diagrams of the CjLGDV (**A**) and AgLGDV (**B**) genomes. Putative open reading frames (ORFs) and their directions are indicated by arrows. Red arrows represent the ORFs having homologous genes in members of the *Naldaviricetes*, and their gene names are labeled in the diagram. Black rectangles represent inverted repeats, gray rectangles represent direct repeats, green rectangles represent PCR confirmed regions, and dark green rectangles represent regions confirmed by Sanger sequencing. The GC content along the genome is displayed in purple. The inner gray-green circle indicates the sequencing coverage depth of the CjLDGV genome. The adenine of the starting codon for the DNA polymerase is set as the starting site for the CjLGDV circular genome. (**C**) Gene-order conservation between CjLGDV/AmFLV and the indicated viruses. Each gene is represented by a dot, with the *x*-axis showing its relative order in the CjLGDV (left) or AmFLV (right) genome, and the *y*-axis indicating the relative position of its homolog in other virus genomes. (**D**) Gene synteny between CjLGDV and AgLGDV. Homologs between CjLGDV and AgLGDV are linked by gray lines. Blue and green ellipses represent genes on the positive and negative strands, respectively.

The unmapped ENLG reads were used for viral genome assembly. After removing low-abundance contigs and assembly of overlapping ones, ambiguous and gap regions were resolved by PCR amplification (Fig. S2) and Sanger sequencing. The primers for the PCR reactions, along with the length and position of the fragments, are listed in Table S1. Finally, we obtained a 142,484 bp circular dsDNA genome (Fig. 2A), which falls within the size range of naldaviricetes. The general features of the GjLGDV, in comparison with representative viruses of the class *Naldaviricetes*, are summarized in Table S2. The GC content in the GjLGDV genome is moderate at 52%. In contrast, most baculoviruses, nudiviruses, hytrosaviruses, and filamentoviruses with similar genome sizes have much lower GC contents.

Given the possibility that CjLGDV may infect multiple ant species, or that related viruses may exist, genetic sequences similar to CjLGDV could be present in ant sequencing data as a result of viral infections. Genome assemblies of 60 ant species from six subfamilies were retrieved from public databases and screened for similar viral sequences by BLAST. Candidate scaffolds representing exogenous viral sequences were expected to fall within the genome size range of naldaviricetes and to display distinct sequence characteristics compared to host genome scaffolds. Finally, a 104,242 bp scaffold (JAPWJP010001941.1) from the long-read sequencing genome assembly of *Anoplolepis gracilipes* (GCA_031304115.1) was identified as a CjLGDV-like sequence. Due to the unavailability of the original sequencing data for this assembly, the presence of the scaffold was then assessed in other publicly available *A. gracilipes* data sets (SRR21231523, SRR21232721, and SRR21232722), and no such sequence was detected. The absence of the scaffold in other sequencing samples suggests that it was derived from virus-infected individuals. Moreover, the scaffold lacks eukaryotic genes and exhibits a GC content distinct from that of host BUSCO scaffolds, further supporting its exogenous viral origin (Fig. S3). Although the sequence may be incomplete, its similar genome size and the presence of conserved genes shared with CjLGDV and members of *Naldaviricetes* suggest that the scaffold contains sufficient genomic information for the virus, which we designate as Anoplolepis gracilipes labial gland disease virus (Fig. 2B).

A TBLASTX similarity assessment revealed that CjLGDV shares more similarity with AgLGDV (22% coverage) and two honey bee filamentous viruses (AmFV, 7% coverage; AmFLV, 4% coverage) than any other viruses (Table S2). A total of 113 and 114 open reading frames (ORFs) with ATG start codons were predicted in the genomes of CjLGDV and AgLGDV, respectively. The average lengths of the predicted ORFs were 351 amino acids (aa) for CjLGDV and 232 aa for AgLGDV, with coding densities of 83.5% and 76.4%, respectively. These genome features are comparable to those of viruses with similar genome sizes in *Naldaviricetes* (Table S2). Among the predicted ORFs, 39 exhibit BLASTP sequence similarities between CjLGDV and AgLGDV, with amino acid identities ranging from 23.9% to 70.7% (Table S3A). Notably, the level of gene synteny between CjLGDV and AgLGDV was higher than that between CjLGDV and other members in *Naldaviricetes* (Fig. 2C and D). These findings suggest that AgLGDV is closely related to CjLGDV.

### Repeat regions as a common feature

Homologous regions (*hrs*), containing repeated sequences composed of imperfect palindrome sequences, are a common feature found in invertebrate dsDNA viral genomes. In baculoviruses, *hrs* function as origins of virus DNA replication and transcription enhancers (23–25). In the CjLGDV and AgLGDV genomes, there are 17 and 14 tandem direct repeat (*dr*) sequences (Table S4), accounting for 4.4% and 3.6% of their genomes, respectively. Repeats in AgLGDV are scattered across the genome, whereas in CjLGDV, six repeats are notably clustered in the region 126,181–131,264 (Fig. 2A; Table S4). All repeats harbor clusters of imperfect palindrome motifs (Fig. S4). These repeats are highly conserved within each viral genome, but no similarities were detected between CjLGDV and AgLGDV or with any other viruses.

Inverted repeat (*ir*) sequences were detected in both viral genomes (Fig. 2A and B; Table S4). The paired repeats exhibit over 98% sequence similarity, with CG content ranging from 46% to 60%. Interestingly, the length of inverted repeats in AgLGDV ranges from 131 to 312 bp, whereas in CjLGDV, repeats are significantly longer, approximately 2,100 bp. The role of these repeats in virus replication remains to be elucidated. In summary, the presence of repeat sequences appears to be a common characteristic of the two ant dsDNA viruses, and the sequences of the repeats are largely virus-specific.

### LGDVs encode conserved core genes shared by viruses of *Naldaviricetes*

Functional annotations of ORFs in the CjLGDV and AgLGDV genomes were conducted by similarity searches based on amino acid sequences and protein structures. In the results, 34 ORFs in CjLGDV (Table S3B) and 29 ORFs in AgLGDV (Table S3C) were found to have homologs in other DNA viruses. The remaining ORFs exhibit either low or no similarity to sequences in available databases. Based on the assumption that viral homologs share similar functions, it was predicted that these genes are involved in DNA replication and processing (*DNApol*, *helicase*, *helicase2*, and *integrase*), transcription and processing (*lef*4, *lef5, lef8, lef9,* and *methyltransferase*), viral packaging and morphogenesis (*ac81*, *p33*, *ATPase*, *trypsin-like serine protease*, and *odv-e18*), viral infectivity (*pif0/p74*, *pif1*, *pif2*, *pif3*, *pif4*, *pif5/odv-e56*, *chitin-binding protein-like*, and *odv-e66*), and apoptosis inhibition (*iap*) (Table S3B and C).

**Fig 3:**
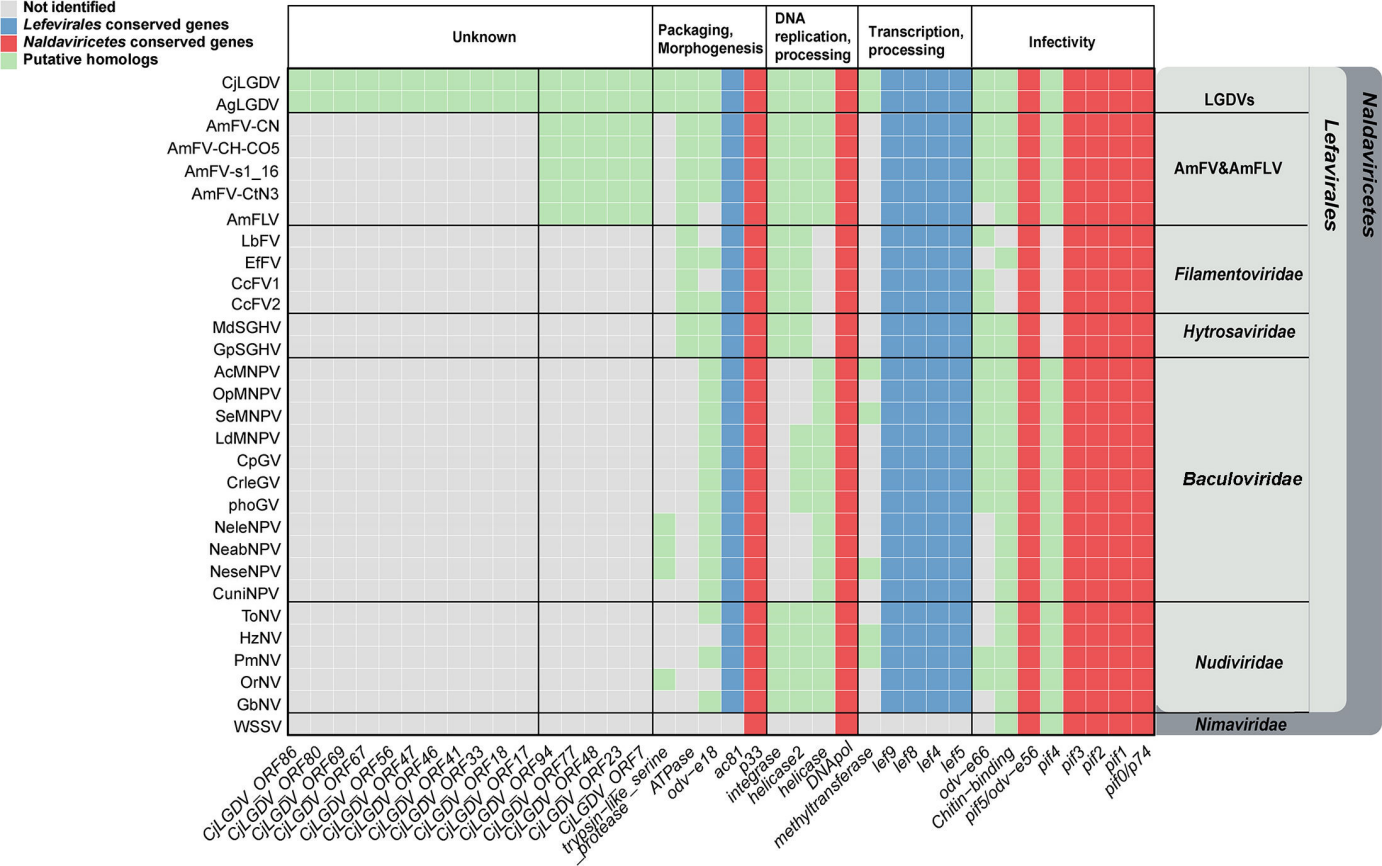
Heatmap of the homologous genes among CjLGDV, AgLGDV, unclassified filamentous viruses, and representative viruses in *Naldaviricetes*. The taxonomic affiliation of the viruses is marked on the right. Rows represent the viral species, and the columns represent the genes grouped by their potential functions. Colored cells indicate the presence of the gene homolog in viral genomes.

Both CjLGDV and AgLGDV genomes contain seven core genes shared by naldaviricetes, including genes for per os infectivity factors (*pif0, pif1, pif2, pif3,* and *pif5*), DNA polymerase gene (*DNApol*), and sulfhydryl oxidase gene (*p33*) (Fig. 3). The *pif* gene family is essential for oral infectivity and is recognized as conserved core genes of naldaviricetes (16). Except for *pif4*, which is absent in hytrosaviruses and filamentoviruses, five *pif* genes (*pif0/p74*, *pif1*, *pif2*, *pif3*, and *pif5/odv-e56*) are present in the genomes of LGDVs as well as in all sequenced viruses within the class *Naldaviricetes*.

Among the annotated genes, four genes (*DNApol*, *helicase*, *helicase2*, and *integrase*) are identified to be involved in viral DNA replication and processing. Both CjLGDV and AgLGDV are predicted to encode a type B DNA polymerase, a common feature of large dsDNA viruses. *helicase* genes, which are commonly found in members of *Lefavirales*, are also present. Similar to nudiviruses, AmFV, AmFLV, and some baculoviruses, two types of *helicase* genes are identified in CjLGDV and AgLGDV genomes. *integrase*, which has been reported to be involved in the excision and circularization of bracovirus DNA, is a conserved core gene in nudiviruses and bracoviruses (26, 27). Here, it is also found in other members of *Lefavirales* except baculoviruses (Fig. 3).

Viruses of *Lefavirales,* comprising the virus families *Baculoviridae*, *Nudiviridae*, *Hytrosaviridae,* and *Filamentoviridae,* are characterized by the possession of conserved baculovirus transcription gene homologs (*lef4*, *lef8*, and *lef9*) and can be phylogenetically distinguished from *Nimaviridae* (16). Five genes (*lef4, lef5, lef8, lef9*, and *ac81*) conserved in lefavirales are detected in both CjLGDV and AgLGDV genomes (Fig. 3). In baculovirus, *lef4*, *lef8*, *lef9,* and *p47* encode the four subunits of viral RNA polymerase (28, 29). The homolog of *p47* is absent in CjLGDV and AgLGDV, a pattern also observed in filamentoviruses, hytrosaviruses, and AmFV/AmFLV (18, 30–33). Additionally, we annotated several previously unidentified *lef* gene homologs in AmFV and AmFLV, including *lef4* in AmFV, and *lef4*, *lef5*, *lef8,* and *lef9* in AmFLV, based on their sequence similarities to CjLGDV (Fig. 3; Table S5).

### Other homologs shared by members of *Naldaviricetes*

CjLGDV and AgLGDV are predicted to encode a FtsJ-like methyltransferase, which is also found in some nudiviruses and baculoviruses (Fig. 3). The methyltransferase is reported to be expressed during the late phase of AcMNPV infection and is involved in the RNA capping process (34). Its removal, however, does not impact the production of budded or occluded viruses in AcMNPV (35). Phylogenetic analysis suggests that viruses may acquire the methyltransferase gene from eukaryotic hosts by horizontal gene transfer (Fig. S5A).

A putative ATPase from the AAA+ superfamily is detected in CjLGDV and AgLGDV, which is also conserved in AmFV, AmFLV, filamentoviruses, and hytrosaviruses (Fig. 3). The AAA+ superfamily of ATPases is widely distributed, where its members participate in diverse cellular processes, including membrane fusion, proteolysis, and DNA replication (36, 37). In baculovirus infection, host ATPases play critical roles in the construction of the viral replication factory and virion morphogenesis (38). Phylogenetic analysis suggests that the viral ATPase was more likely acquired somehow from bacteria other than their eukaryotic host by horizontal transfer (Fig. S5B).

Insect DNA viruses commonly interfere with the host immune system by preventing apoptosis. Both CjLGDV and AgLGDV encode proteins containing the baculoviral IAP repeat domain, predicted to be homologs of inhibitors of apoptosis (IAPs). IAPs are anti-apoptotic regulators that prevent apoptosis by inhibiting the caspase family of proteases, and they are widely distributed in large dsDNA viruses, yeast, nematodes, insects, and mammals (39). Phylogenetic analysis indicates that the LGDVs *iap* genes may have eukaryotic origins (Fig. S5C).

Both CjLGDV (*orf81*) and AgLGDV (*orf4*) are predicted to encode a protein containing a chitin-binding domain. Similar domains are also found in proteins of entomopoxviruses and baculoviruses, where they are known to play critical roles in oral infectivity (40, 41). However, the LGDV homologs, especially CjLGDV ORF81, have larger molecular weights and more complex overall structures than AcMNPV chitin-binding proteins (Ac145 and Ac150) (Fig. S6), suggesting that they may perform additional or distinct functions.

Several multigene families are identified in CjLGDV and AgLGDV genomes. Three Baculovirus Repeated ORF (BRO) homologs with an N-terminal DNA-binding domain are detected in CjLGDV (Table S3B). The *bro* genes are prevalent in various viruses in the *Naldaviricetes* class, with baculoviruses containing 0–16 *bro* genes. However, the specific functions of these genes still remain unknown.

Two ORFs encoding putative trypsin-like serine proteases are conserved in CjLGDV and AgLGDV. Trypsin-like serine proteases are enzymatic proteins commonly found in various RNA and DNA viruses as well as in cellular organisms (2). Typically classified as nonstructural proteins in many viruses, these proteases play an indispensable role in viral maturation by facilitating proteolysis through serine-type endopeptidase activity (42, 43).

Two baculovirus envelope structural protein homologs (ODV-E18 and ODV-E66) are detected in CjLGDV and AgLGDV. *odv-e18* is one of the conserved core genes in baculoviruses and is essential for the budded virus production (44). *odv-e66*, identified in alphabaculovirus and betabaculovirus that infect Lepidoptera hosts, is also present in hytrosaviruses, AmFV, certain filamentoviruses, and nudiviruses. The copy number of *odv-e66* varies among viruses, with most containing 0–5 copies. However, in bracoviruses, it has expanded to 36 genes distributed across 10 genomic regions, likely playing a critical role in wasp adaptation (45). In baculoviruses, ODV-E66 is a major envelope protein with chondroitinase activity that degrades the larval peritrophic membrane, facilitating oral infection (46). In the CjLGDV and AgLGDV genomes, two adjacent *odv-e66* homologs are annotated (Fig. 2A and B). Phylogenetic analysis indicates that ant virus *odv-e66* homologs cluster with those of naldaviricetes, forming a monophyletic subclade from bacteria, suggesting that these viral *odv-e66* genes might be acquired by an ancestral virus from bacteria (Fig. S7A). Additionally, we found that the viral ODV-E66 homologs share a conserved domain with bacterial chondroitin lyases, including three conserved amino acids previously identified crucial for the enzyme activity in bacteria and baculoviruses (47, 48) (Fig. S7A). Of the two ODV-E66 homologs, ODV-E66a retains all three conserved amino acids, while ODV-E66b is mutated at two of the three sites (N to D and H to R). The mutations at the conserved sites are predicted to cause some structural changes in the enzyme activity center, which may affect the function of the protein (Fig. S7B).

### Conserved genes shared exclusively by LGDVs and AmFV/AmFLV

Eleven genes (*CjLGDV_orf17*, *18*, *33*, *41*, *46*, *47*, *56*, *67*, *69*, *80*, and *86*) are uniquely shared by CjLGDV and AgLGDV, and they show no significant similarity to any genes in other known viruses (Fig. 3). In addition, five conserved genes in LGDVs (*CjLGDV_orf7*, *23*, *48*, *77*, and *94*) are also found in all AmFV and AmFLV, but they have no homologs in other viruses. For all the conserved genes exclusively shared by LGDVs and AmFV/AmFLV, no significant sequence or structural similarity to known proteins was detected, preventing reliable functional annotation. Nevertheless, their conservation in LGDVs, and some also in AmFV and AmFLV, suggests that they may play important roles in the unique biology of these viruses.

### Phylogenetic position of LGDVs

**Fig 4:**
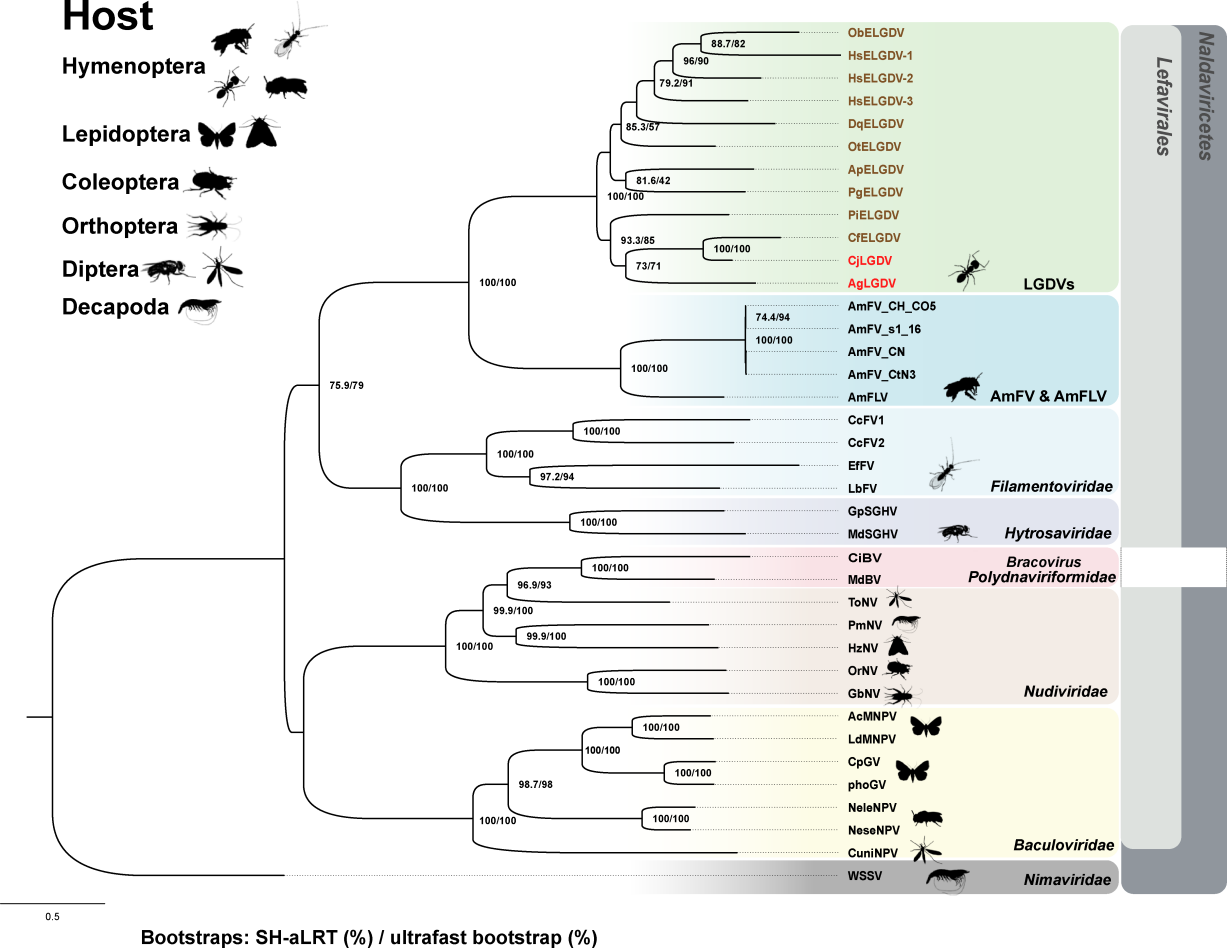
Phylogeny of LGDVs with viruses in *Naldaviricetes*. The phylogenetic tree was constructed using maximum likelihood inference based on concatenated amino acid sequences of 12 conserved genes (*p74*, *pif1*, *pif2*, *pif3*, *pif5*, *lef4*, *lef5*, *lef8*, *lef9*, *DNApol*, *p33,* and ac*81*). Gene accession numbers are provided in Table S5. Node support values are indicated as SH-aLRT support (%)/Ultrafast bootstrap (%). The scale bar represents the average number of amino acid substitutions per site across the tree. Each viral family is denoted by a unique color, and icons next to each virus name indicate the arthropod order of the respective host.

CjLGDV shares several key characteristics with the viruses in *Naldaviricetes*, including their infection of arthropod hosts, the presence of long filamentous nucleocapsids in an envelope, a large circular dsDNA genome, and conserved core genes. These shared traits strongly support the classification of LGDVs as a member of *Naldaviricetes*. To elucidate the phylogenetic relationships of CjLGDV and AgLGDV, a highly supported phylogenetic tree was generated using the maximum likelihood method, based on the concatenated alignment of the 12 genes conserved in lefavirales (Fig. 4). In this tree, the interrelationships between *Naldaviricetes* members are consistent with previously reported results (27, 33, 49). CjLGDV and AgLGDV cluster together, occupying a unique position within the phylogenetic tree. They are grouped with AmFV and AmFLV, forming a distinct clade adjacent to filamentoviruses and hytrosaviruses, but far from baculoviruses and nudiviruses.

Evolutionary distances within and between virus families in the *Lefavirales* were calculated based on the 12 genes conserved in lefavirales (Fig. S8). As expected, patristic distances within families are smaller (0.49–2.85) than those between families (3.08–4.38). The distance between CjLGDV and AgLGDV (1.14) falls within the range observed for intrafamily distances, being significantly lower than interfamily distances. Interestingly, the distances between honeybee filamentous viruses (AmFV and AmFLV) and LGDVs (2.49–2.71) are close to the upper limit but still fall within the range of intrafamily distances, suggesting a close evolutionary relationship.

Overall, the phylogenetic analysis reveals a close relationship between CjLGDV and AgLGDV, and they may represent members of a novel virus family in the class *Naldaviricetes,* order *Lefavirales*.

### Endogenous viral elements in ant genomes

Previous studies have demonstrated a rich diversity of EVEs in ant genomes, highlighting their evolutionary and functional significance (12, 14). To expand our understanding of ant EVEs, we performed similarity searches against the genomes of 60 ant species available in NCBI databases, using the CjLGDV genome as a query.

Although the detection and characterization of EVEs in some species are constrained by the quality and completeness of available genome assemblies, a total of 1,844 loci were identified as candidate EVEs across the genomes of 36 ant species from five subfamilies, including *Myrmicinae*, *Formicinae*, *Ponerinae*, *Dolichoderinae,* and *Pseudomyrmicinae* (Table S6). Of these, 1,407 loci were considered high-confidence endogenous sequences (Fig. S9; Table S6). In a previous comprehensive study of insect EVEs, 278 high-scoring AmFV-related EVEs were identified in ants (14). Among these, 248 were re-identified in our results (Table S6), highlighting the sensitivity and accuracy of the detection pipeline.

**Fig 5:**
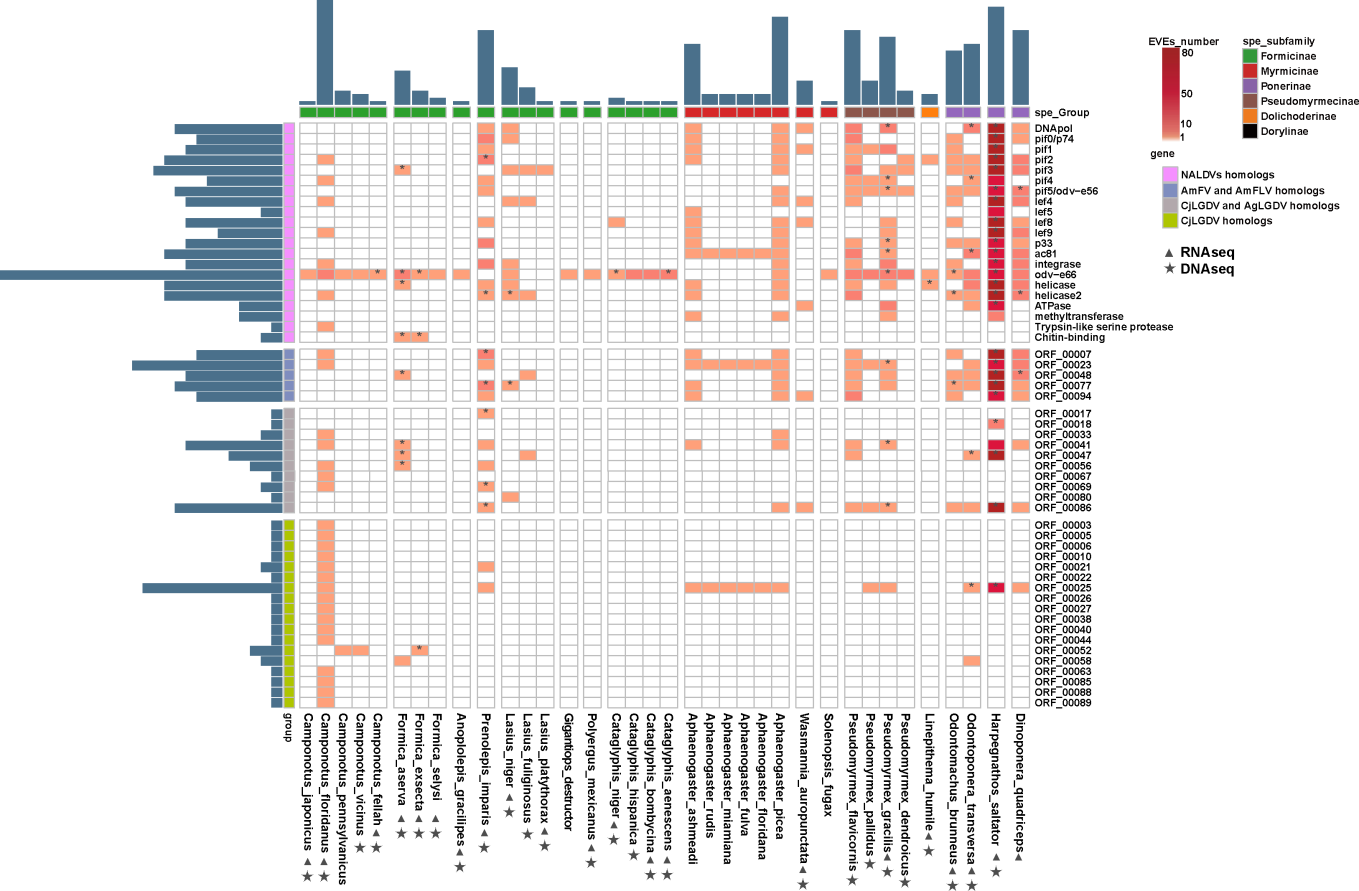
CjLGDV-related endogenous viral elements in ant genomes. Endogenous viral elements identified in the genomes of 36 ant species are shown in a heatmap. Orange cells indicate the presence of the viral homolog in ant genomes. Rows represent the viral homologs, and columns represent the ant species. Bar plots adjacent to the heatmap represent the number of homolog types in each ant (top) and the number of ant species containing each viral homolog (left). Cells marked with * indicate that the gene expression has been detected in the ant. Filled triangle and star under the ant names, respectively, indicate that raw RNA seq and DNA seq data of that species are available in databases.

The EVEs identified in this study exhibit homology to 54 CjLGDV genes, including 21 genes with homologs in viruses of the class *Naldaviricetes*, 5 genes with homologs in AmFV and AmFLV, 10 genes with homologs in AgLGDV, and 18 genes unique to CjLGDV (Fig. 5). BLASTP analysis revealed amino acid sequence identities between the EVEs and viral homologs, ranging from 22% to 76.9% (Table S6). Phylogenetic analyses demonstrated a closer relationship between these EVEs and LGDVs than with AmFV, AmFLV, or other known viruses (Fig. 4; Fig. S101–28). These EVEs are distributed across various scaffolds in ant genomes, with scaffold sizes ranging from hundreds of base pairs to hundreds of millions of base pairs (Fig. S11). Each scaffold contains between 1 and 37 viral homologs.

Among the ant species analyzed, the highest abundance of EVEs was detected in *Harpegnathos saltator*, where 1,378 loci spread across 174 scaffolds showed homology to 29 CjLGDV genes. These homologs are represented by numerous paralogs in the *H. saltator* genome (Fig. 5), consistent with a previous observation (14). Phylogenetic analyses grouped these paralogs into three distinct clusters (Fig. S12A). Within each cluster, paralogs have low patristic distances (0.01–0.4) (Fig. S12B), share high sequence identity (81%–100%) (Fig. S12C), and have relatively conserved flanking regions (Fig. S12D). Comparative analysis of host gene content between EVE-containing scaffolds revealed distinct sets of eukaryotic genes (Fig. S13). These findings suggest that *H. saltator* experienced at least three independent viral endogenization events, each followed by lineage-specific duplications that expanded the copy number of the integrated viral sequences.

In *Camponotus floridanus*, a distinct EVE pattern was observed. Across three scaffolds, 37 loci showed homology to 31 CjLGDV genes, with 35 concentrated on one scaffold with a length of about 100 kb (NW_020229367.1). These loci were confirmed as endogenous fragments based on several lines of evidence: the presence of identical sequences in another *C. floridanus* genome assembly (GCA_000147175.1) and the occurrence of multiple premature stop codons and transposable elements within these regions. RNA-seq analyses across multiple data sets did not detect any transcriptional activity in the EVE regions in *C. floridanus*.

Gene synteny analysis of five representative EVEs containing multiple viral gene homologs revealed that all the EVEs had some degree of gene collinearity with CjLGDV (Fig. S14A), supporting the evolutionary linkage between CjLGDV and the ant hosts. The 100 kb long region within the scaffold (NW_020229367.1) in the *C. floridanus* genome showed high synteny with the CjLGDV genome (Fig. S14B), suggesting that this scaffold was very likely derived from a virus closely related to CjLGDV.

To investigate the selective pressures acting on ant EVEs, we analyzed the ratio of nonsynonymous to synonymous substitution rates (*d_N_*/*d_S_*) in the genes that appeared at least twice in the ant EVEs identified in this study. The results revealed that most CjLGDV homologs in ant EVEs have undergone strong purifying selection (*d_N_*/*d_S_* < 1; Fig. S15; Table S6). By searching available RNA-seq data, it was shown that at least 135 EVEs in 13 ant species were transcriptionally active (Table S6), of which 67 exhibited *d_N_*/*d_S_* < 1, suggesting evolutionary constraints on these EVEs and their potential functional importance.

## DISCUSSION

Substantial progress has been made in elucidating the viral landscape in invertebrates (2, 50), with various insects identified as hosts to large DNA viruses (18, 19, 30, 32, 49, 51). However, few DNA viruses infecting ants have been characterized so far (6, 7, 11). This study presents the first fully sequenced dsDNA virus from ants, which is tentatively named Camponotus japonicus labial gland disease virus. Although high copy numbers of viral genomes, especially RNA viruses, have been reported in some arthropods without obvious pathology (52–55), our findings suggest a potential association between CjLGDV and labial gland disease. Specifically, in individuals with visibly enlarged labial glands, we observed the presence of abundant viral particles and a high ratio of DNA sequencing reads (41.20% of CjLGDV vs 48.90% of ant host). While only a subset of ants harboring the virus showed gland enlargement, all ants with enlarged labial glands were detected to be infected with the virus. These observations indicate that high viral load may be correlated with labial gland enlargement.

The observation of elongated nucleocapsids stacked in the nucleus, along with coiled and uncoiled nucleocapsids covered with membrane in the cytoplasm, suggests a lifecycle of CjLGDV similar to that of most DNA viruses that replicate in the nucleus and mature in the cytoplasm. By sequence assembly, it is revealed that CjLGDV contains a 142 kb circular dsDNA genome. In addition, mining of publicly available genomic resources in this study uncovered a 104 kb CjLGDV-like scaffold in *Anoplolepis gracilipes*, which displays high gene synteny with the CjLGDV genome and is named AgLGDV. These findings point to the existence of a previously unrecognized family of large DNA viruses infecting ants.

CjLGDV and AgLGDV exhibit notable genomic similarities to viruses in the class *Naldaviricetes*, including comparable GC content, genome size, coding density, and the presence of repetitive sequences. The presence of conserved *Naldaviricetes* gene homologs in the LGDVs genomes supports their classification within this group. Although viruses in the class *Naldaviricetes* generally share similar genome sizes, LGDVs, filamentoviruses, hytrosaviruses, and AmFV have significantly longer nucleocapsids than baculoviruses and nudiviruses. Moreover, occlusion bodies, which are critical for oral infectivity of baculoviruses, are absent in LGDVs, filamentoviruses, hytrosaviruses, and AmFV.

The identification of *pif* gene homologs and three DNA-directed RNA polymerase subunits (*lef4*, *lef8*, and *lef9*) suggests that LGDVs are affiliated with the order *Lefavirales*. Phylogenetic analysis of 12 conserved genes in naldaviricetes positions CjLGDV and AgLGDV within a novel lineage of the order *Lefavirales*. These two ant viruses form a monophyletic clade with the unclassified bee-infecting viruses AmFV and AmFLV, whereas filamentoviruses and hytrosaviruses constitute a closely related sister group, and baculoviruses and nudiviruses are in distant branches on the phylogenetic tree. Notably, phylogenetic analysis of the ATPase gene, which is conserved across LGDVs, filamentoviruses, hytrosaviruses, and AmFV/AmFLV, suggests that this gene was very likely acquired from bacteria through a single ancestral horizontal gene transfer event, further reinforcing the shared evolutionary ancestry among these viral groups. Among these viruses, LGDVs also share a greater number of homologous genes and exhibit higher sequence identity with AmFV/AmFLV than with other members of *Lefavirales*, indicating that they have a closer evolutionary relationship.

However, there also exist some important distinctions between LGDVs and AmFV/AmFLV. First, the genome sizes of LGDVs (142 kb for CjLGDV and 104 kb for AgLGDV) are much smaller than those of AmFVs (close to 500 kb). Second, while both CjLGDV and AmFV possess long filamentous nucleocapsids, the characteristic three figure-eight looped nucleocapsids observed in AmFV virions are absent in CjLGDV (18, 56–58). Third, only about one-fifth of the ORFs encoded by LGDVs have homologs in AmFV/AmFLV, and the genome sequence similarities between LGDVs and AmFV/AmFLV were clearly below 10%. The discovery of more viruses closely related to LGDVs and AmFV/AmFLV would be helpful to elucidate their evolutionary relationship and establish the taxonomy of these unclassified viruses.

The close relationship of LGDVs to AmFV/AmFLV raises intriguing questions about their evolutionary trajectory. Considering the intersecting ecological niches and interspecies interactions between ants and bees, ants have been proposed as potential vectors or reservoirs for pathogens that affect bee populations (59–64). It is thus plausible that host ecology has played a role in shaping the co-evolution and cross-species transmission of these large DNA viruses. The enlarged labial gland observed in *C. japonicus* is reminiscent of the salivary gland hypertrophy seen in flies infected with hytrosaviruses (22, 65), probably indicating a shared pathological mechanism with a common evolutionary origin. Hytrosavirus-like viral genome fragments have been detected in *Gigantiops destructor* (6)*,* indicating the possibility of hytrosavirus infection within ants and a potential connection between LGDVs and hytrosaviruses. Hytrosavirus has been considered an attractive candidate for fly biocontrol as the virus infection can reduce vitellogenesis and disrupt mating behavior in the infected host. The identification of a novel DNA virus AgLGDV in *Anoplolepis gracilipes*, one of the most damaging invasive tramp ants globally, provides a potential candidate for its biocontrol. Future studies on the virus infection and its histopathological effects on the invasive host will be essential to evaluate its feasibility, efficacy, and safety as a biocontrol agent. LbFV is associated with the superparasitism behavior of *Leptopilina boulardi* (66, 67), and the virus infection may contribute adaptive genes to parasitic wasps (68, 69). Viral manipulation of host behavior represents a sophisticated evolutionary strategy that optimizes transmission conditions and enhances survival, illustrating the complex interplay between virus and host (70, 71). Further research is required to determine whether LGDVs induce behavioral modifications in ants, which could deepen our understanding of host-pathogen dynamics.

EVEs are the result of chromosomal integration of viral genes in the host germline cells. Non-retroviral EVEs represent rare remnants of ancient viral infections. Studying these EVEs can provide valuable insights into viral host range, ancestral viral diversity, and the timing of viral evolutionary events. In this study, an unprecedented number of EVEs, which were more closely related to CjLGDV and AgLGDV rather than AmFV or any other known viruses, were detected in the genomes of various ant species spanning five subfamilies (*Myrmicinae*, *Formicinae*, *Ponerinae*, *Dolichoderinae,* and *Pseudomyrmicinae*). Given the broad detection of EVEs in ants and the low probability of non-retroviral endogenization events occurring in the germline, this finding indicates prolonged and frequent interactions between the ancestral LGDVs and their hosts. The discovery of CjLGDV in *C. japonicus* and AgLGDV in *A. gracilipes*, along with reports of “labial gland disease” in various ant species, implies that such interactions have persisted in their descendants. Some EVEs identified in this study exhibit high gene collinearity and close phylogenetic relationships with CjLGDV, suggesting that some recent integration events or integrations from more closely related viruses have occurred. Furthermore, abundant EVEs and multiple independent endogenization events detected in *H. saltator* provide strong evidence of long and frequent interactions between ants and viruses. All these findings support the hypothesis of long-term and frequent interactions between LGDVs and ants. Further efforts on the direct detection and characterization of viruses from ants are necessary to conclusively establish the natural host range of LGDVs and to elucidate the relationship between LGDVs and LGDVs-related EVEs.

Studies have shown that EVEs can serve as reservoirs of immune memory in hosts and may function in antiviral defense (72–75). In some parasitic wasps, endogenous viral elements have been discovered to produce “virus-like structures” in females’ reproductive organs, which can deliver immunosuppressive DNA or proteins to modulate the immune system of their hosts (69, 76–81). In our study, viral homologs in CjLGDV-related EVEs are found to be under strong purifying selection, and the transcription of some EVEs in some ant species can be spotted from the publicly available databases. These findings suggest the potential domestication of CjLGDV-related EVEs for functional roles. One such gene, *odv-e66,* which encodes a chondroitinase associated with viral infectivity, has been hypothesized to be a key factor in virus-host adaptation (45). Here, we reveal that homologs of *odv-e66* are widely distributed in diverse ant species, and some of them are transcriptionally active. Whether ODV-E66 plays a functional role and contributes to ant evolution remains a question that requires further investigation.

Taken together, we identified two novel dsDNA viruses, CjLGDV and AgLGDV, in ants. Genomic features and phylogenetic analysis of the two viruses suggest that they belong to a new viral family in the order *Lefavirales*, class *Naldaviricetes*. The detection of LGDV-related endogenous viral elements in various ant genomes provides evidence that ants have been the host to LGDVs or their ancestors for a long time. These findings expand the known diversity of naldaviricetes, broaden our understanding of their host range, and reveal a long and frequent interaction between LGDVs and ant hosts.

## MATERIALS AND METHODS

### Ant collection

Colonies of *Camponotus japonicus*, in which minor workers were observed to have enlarged labial glands (22), were collected from Yangling, Shaanxi Province. The ants were refrigerated at −20°C for 10 minutes to reduce their activity, after which their labial glands were dissected in Ringer’s physiological solution under an Olympus SZ51 microscope.

### Electron microscopy

Labial glands were initially fixed in 2.5% cold glutaraldehyde for at least 12 hours, then washed five times with PBS buffer (0.1 M, pH 7.2). Post-fixation treatment was performed in 1% osmium tetroxide for 2 hours, followed by five washes with PBS. Samples were dehydrated through a graded ethanol series (30%, 50%, 70%, 80%, 90%, and 100%) and 100% acetone. The tissue was infiltrated with LR-White resin and acetone mixtures (1:3 for 2 hours, 1:1 for 5 hours, and 3:1 for 12 hours), then embedded in pure LR-White resin and polymerized over a period of 72 hours. The thin sections were obtained using a Leica EM UC7 ultramicrotome (Hitachi, Tokyo, Japan), and double-stained using uranyl acetate for 20 minutes followed by lead citrate for 10 minutes to enhance contrast. Observation of the stained sections was conducted using a Tecnai G2 Spirit Bio Twin electron microscope (FEI, Czech Republic and USA).

### Genomic DNA preparation and sequencing

Normal labial glands and enlarged labial glands were obtained from ants in the same colony. The genomic DNA was extracted from the entire labial glands with a genomic DNA extraction kit (BioTeke, Beijing, China) following the manufacturer’s protocol. The quantity and quality of extracted DNAs were measured using a NanoDrop ND-1000 spectrophotometer (Thermo Fisher Scientific, Waltham, MA, USA) and agarose gel electrophoresis, respectively. The extracted DNA was processed to construct metagenome shotgun sequencing libraries with an insert size of 400 bp by using Illumina TruSeq Nano DNA LT Library Preparation Kit. The library was sequenced on the Illumina HiSeq X-ten platform (Illumina, USA) with a PE150 strategy by Personal Biotechnology Co., Ltd. (Shanghai, China).

### Viral genome assembly

Paired-end sequencing reads were quality-filtered and trimmed using Cutadapt version 4.4 (82). Genomes of three species closely related to *Camponotus japonicus*—*Camponotus floridanus*, *Camponotus pennsylvanicus,* and *Camponotus vicinus*—were merged into a composite reference genome. Sequencing data from the LG and ENLG groups were aligned to the reference using Bowtie2 version 2.5 (83). Reads that aligned to the reference genome were regarded as host sequences, and the remaining reads were utilized for viral genome assembly using Haploflow (84). The quality of the assembly was evaluated using QUAST version 5.2 (85). All reads used for genome assembly were mapped to the assembled contigs using Bowtie2, with coverage determined by bedtools version 2.31 (86). Low-abundance contigs were filtered out, and overlapping ones were merged. To resolve gaps and ambiguous regions between contigs, PCR was performed using primers listed in Table S1, and the sequences of the amplified fragments were determined by Sanger sequencing.

### Sequence analyses and genome annotation

Whole-genome similarities were evaluated by employing TBLASTX (87, 88) against a set of representative invertebrate DNA virus genomes. Putative open reading frames (ORFs) were identified using ORF finder and Prodigal version 2.6 (89). ORFs were named based on their homologs or genomic location. BLASTP was used to identify ORF similarities against the NCBI nonredundant protein database. Domain identification within ORFs was performed using the NCBI Conserved Domain Search (90) and HMMER search against both public databases (CDD and PFAM) and local databases. The local databases were built using homologous sequences from nuclear arthropod large DNA viruses, which were aligned using MAFFT version 7.5 (91) and converted into HMMs using hmmbuild. Protein structure alignment and homologous structure searches were conducted using Foldseek (92). The sequence coding density was calculated as the ratio of the base number of all ORFs to the base number of the genome. Tandem direct repeats and imperfect palindromic motifs were identified using the etandem (93) and MEME suite (94), respectively, with a 100 score cutoff. The virus genome was graphically represented in a circular diagram using CGView (95).

### Phylogenetic analysis

The phylogenetic position of CjLGDV within *Naldaviricetes* was inferred by a maximum likelihood tree based on 12 conserved genes (*p74*, *pif1*, *pif2*, *pif3*, *pif5*, *lef4*, *lef5*, *lef8*, *lef9*, *DNApol*, *p33*, and *ac81*). Sequence accession numbers for the conserved genes used in the analysis are provided in Table S5. Specifically, amino acid sequences were aligned using MAFFT with the E-INS-I mode. These alignments were subsequently trimmed by trimAl version 1.4 (96) and concatenated into a single protein alignment by SequenceMatrix (97). Phylogenetic trees were then constructed using the maximum-likelihood method implemented in IQ-TREE version 2.2 (98). ModelFinder (99) was employed within IQ-TREE to identify the best models for each partition. White spot syndrome virus was selected as the outgroup. Node supports in the ML trees were determined using Ultra-fast bootstrap (100) and SH-aLRT (options -bb 1,000 and -alrt 1,000). The patristic distances within and between viral families were performed by the ape R package (101).

### Identification of endogenous viral elements

A BLAST-based approach complemented by systematic phylogenetic clade validations was utilized to identify endogenous viral sequences. Putative protein sequences of CjLGDV were subjected to a TBLASTN search against a database comprising 60 ant genomes. Information on the ant genomes is provided in Table S7. Only hits with an e-value smaller than 1e-5 and more than 20% sequence alignment coverage were maintained. Adjacent hits within a distance of 10 bp were merged into a single entity. Hits were then subjected to a BLASTX search against the NCBI nonredundant protein database. Sequences that clearly align with viral sequences were identified as potential EVEs. Several criteria were utilized to evaluate the endogenous characteristics of candidate EVEs, including the presence of transposable elements, insect genes, premature stop codons, and sequencing depth.

To assess the authenticity of putative EVEs identified in ant genomes, we implemented a quantitative scoring scheme based on multiple genomic features indicative of viral integration. Each EVE was assigned points according to the following criteria: 2 points for the presence of premature stop codons in EVEs, suggesting non-functional or degraded viral sequences; 1 point if the sequencing depth was comparable to that of host scaffolds; 1 point for the presence of annotated eukaryotic genes on the scaffold; 1 point for the presence of transposable elements; 1 point for a GC content similar to that of host scaffolds; 1 point if the scaffold length substantially exceeded that of known exogenous *Naldaviricetes* viral genomes (>500 kb). Based on the total score, EVEs were classified as high confidence (>4 points), medium confidence (3–4 points), or low confidence (≤2 points).

Transposable elements on the scaffolds were predicted using EDTA version 2.0 (102), while genes were predicted using AUGUSTUS version 3.5 (103). Taxonomic assignments were performed on sequence similarity with the Uniprot/Swissprot database by BLASTP. Only genes assigned to insects were retained. Details on these EVE sequences are available in Table S6. To perform phylogenetic analyses, EVEs were aligned with related homologs from CjLGDV and NALDVs using MAFFT version 7.5 and subsequently refined with trimAl version 1.4. Phylogenetic trees were constructed using the maximum-likelihood method implemented in IQ-TREE version 2.2. To assess potential functional constraints on the EVEs, the ratio of nonsynonymous substitution rate (*d_N_*) to synonymous substitution rate (*d_S_*) was estimated using codeml on the PAML package (104).

## Data Availability

The data underlying this article are available in the article and in its online supplemental material. The CjLGDV genome sequence has been deposited in the GenBank Nucleotide database under accession number PP861292. The DNA sequencing data generated in this study have been deposited in the NCBI SRA database under accession number PRJNA1119426.

## SUPPLEMENTAL MATERIAL

Supplemental figures jvi.01178-25-s0001.pdf Figures S1 to S15.

Table S1 jvi.01178-25-s0002.xlsx Primers used for virus genome assembly.

Table S2 jvi.01178-25-s0003.xlsx General features of CjLGDV genome in comparison with other dsDNA viruses.

Table S3 jvi.01178-25-s0004.xlsx BLASTP results between CjLGDV and AgLGDV, and ORFs in CjLGDV and AgLGDV with homologs in large dsDNA viruses infecting arthropods.

Table S4 jvi.01178-25-s0005.xlsx Repeats in virus genomes.

Table S5 jvi.01178-25-s0006.xlsx Information on the conserved genes of CjLGDV and AgLGDV with putative homologs in Naldaviricetes.

Table S6 jvi.01178-25-s0007.xlsx Information on CjLGDV-related endogenized loci in ant genomes.

Table S7 jvi.01178-25-s0008.xlsx Information of the ant genomes used in this study.

ASM does not own the copyrights to Supplemental Material that may be linked to, or accessed through, an article. The authors have granted ASM a non-exclusive, world-wide license to publish the Supplemental Material files. Please contact the corresponding author directly for reuse.
