# Supplementary material for: Discovery of a novel double-stranded DNA virus associated with ant labial gland disease reveals its long-term interaction with ants
Source: J Virol. 2025 Sep 17;99(10):e01178-25. doi: 10.1128/jvi.01178-25 (PMC12548425; doi:10.1128/jvi.01178-25)
Supplement: Supplemental figures — Figures S1 to S15. [file jvi.01178-25-s0001.pdf]

Supplementary figures for

Discovery of a Novel Double-Stranded DNA Virus Associated with Ant Labial Gland Disease Reveals its Long-term Interaction with Ants

Shengqiang Jiang[a]#, Liangliang Zhang[b]#, Xingyu Guo[a], Jianchao Li[a], Jing Hu[a], Hong He[b]*, Hongying Chen[a,c]*

[a] College of Life Sciences, Northwest A&F University, Yangling, Shaanxi, 712100, P. R. China

[b] Key Laboratory of National Forestry and Grassland Administration for Control of Forest Biological Disasters in Western China, College of Forestry, Northwest A&F University, Yangling, Shaanxi, 712100, China

[c] Institute of Future Agriculture, Northwest A&F University, Yangling, Shaanxi, 712100, China.

# These authors contributed equally to the work.

* Correspondence: Hongying Chen, chenhy@nwsuaf.edu.cn (H.C.); Hong He, hehong@nwsuaf.edu.cn (H.H.).

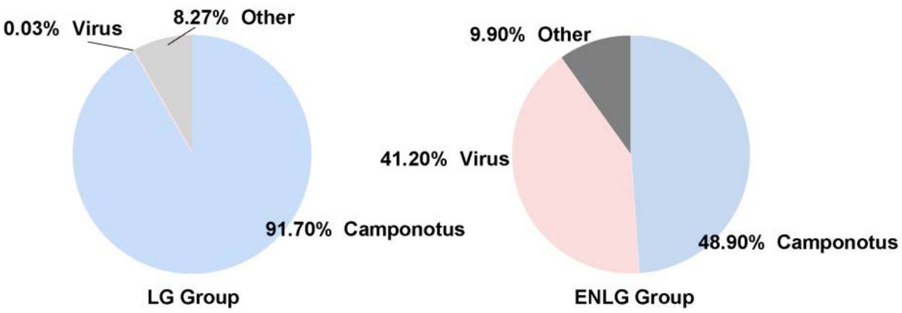

Supplementary Figure S1 Mapping rate of the deep sequencing reads. ENLG, enlarged labial gland; LG, normal labial gland. Camponotus, for reads mapped to the host genome; Virus, for reads mapped to the CjLGDV genome assembled in this study; other, for unmapped reads.

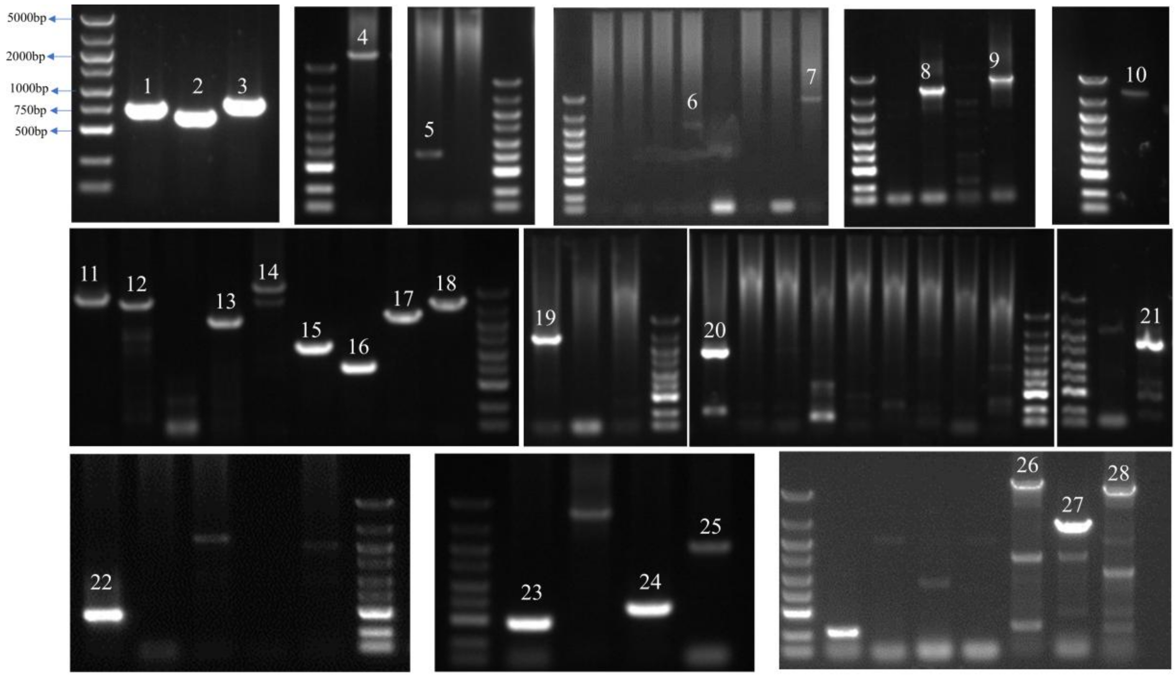

Supplementary Figure S2 Completion and confirmation of CjLGDV assembly by PCR amplification. Primers used for the amplification of the numbered fragments are listed in supplementary table S1. The sequences in fragments 1-12 and 19-22, which covered all the ambiguous regions from the high throughput sequencing, were verified by Sanger sequencing.

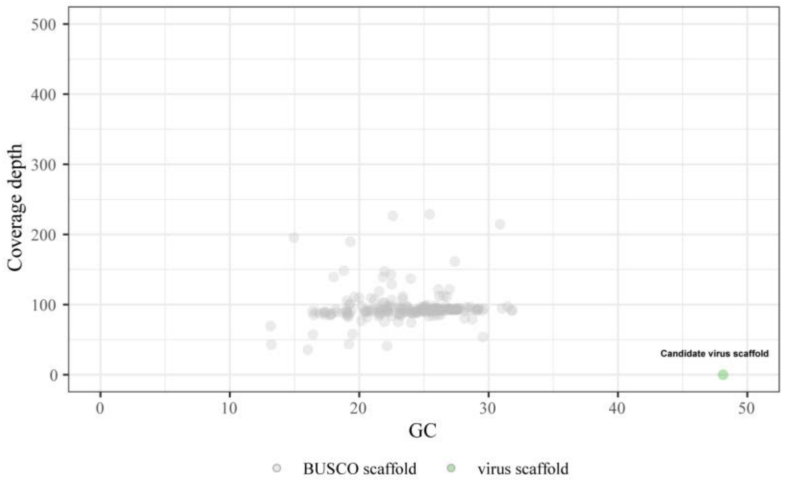

Supplementary Figure S3 Differences in CG content and sequencing coverage depth between ant host BUSCO scaffolds of *Anoplolepis gracilipes* (GCA_031304115.1) and the viral scaffold (JAPWJP010001941.1) found in the ant sample. The CG content and sequencing coverage depth of ant host BUSCO scaffolds are represented by grey dots, while the viral scaffold is represented by a green dot.

Figure S4A: AgLGDV

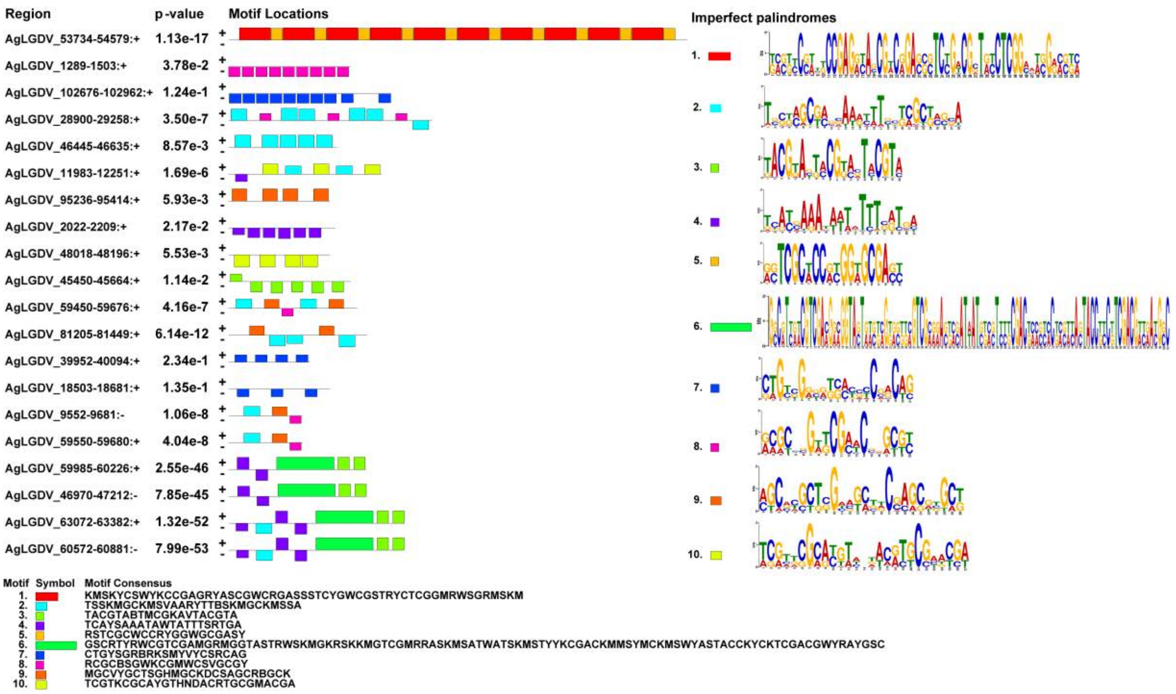

Figure S4B: CjLGDV

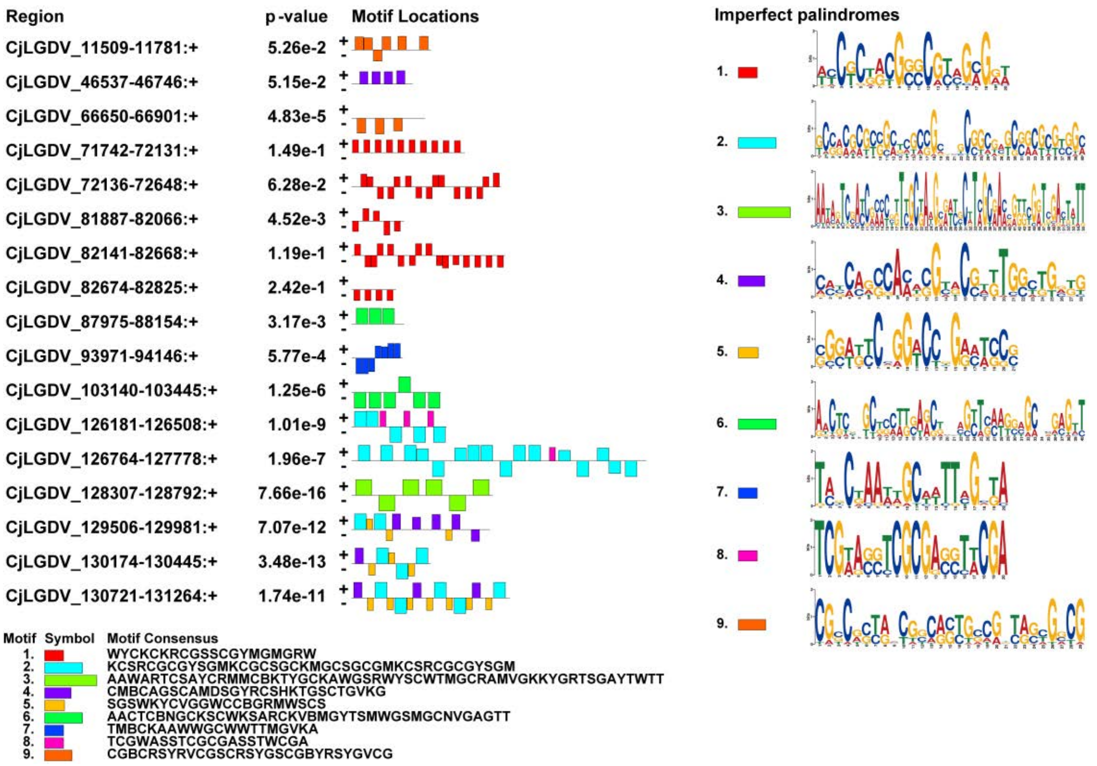

Supplementary Figure S4 Imperfect palindromic motifs. Details of imperfect palindromic motifs in repeat sequences in the CjLGDV and AgLGDV genomes.

Figure S5A Methyltransferase

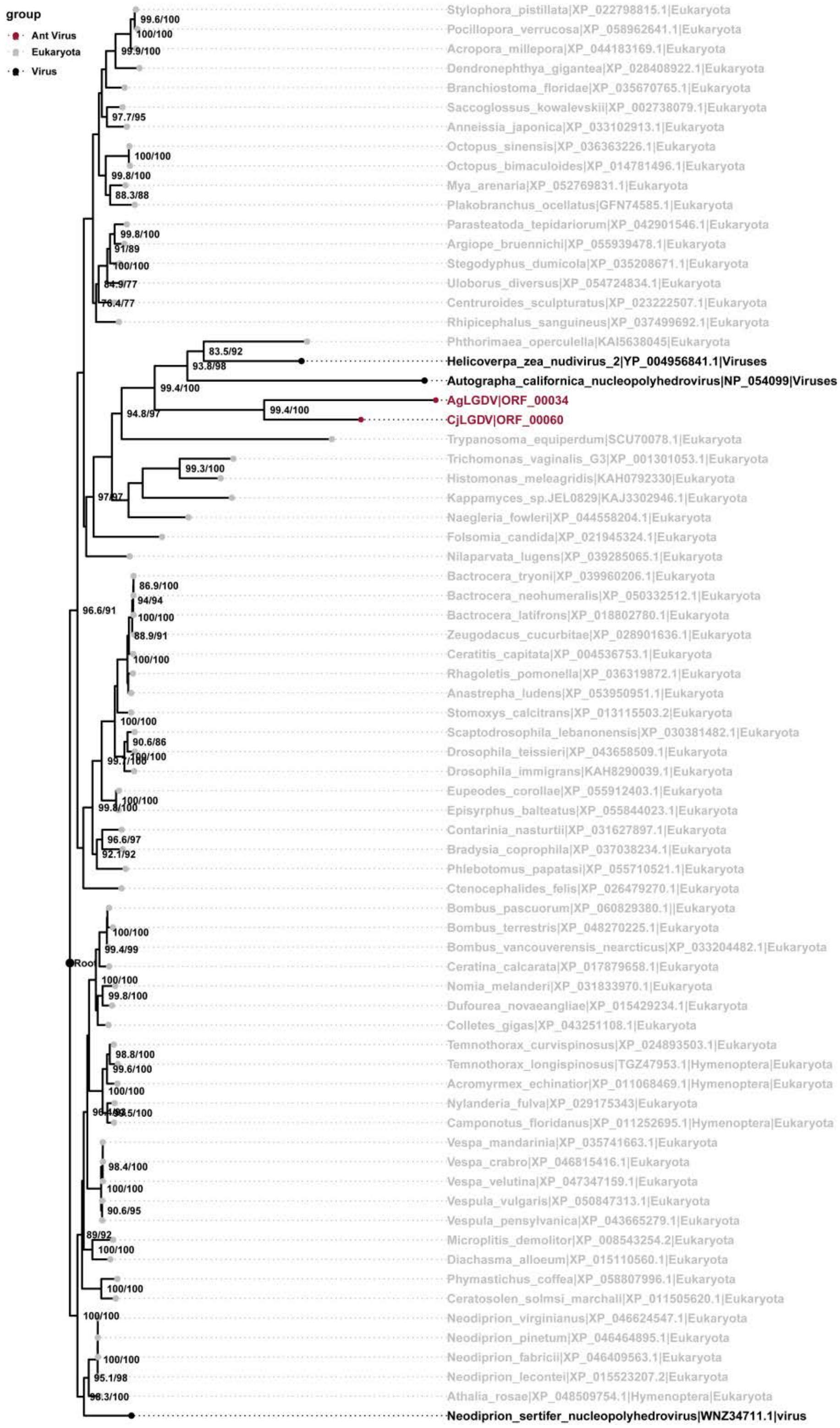

Figure S5B ATPase

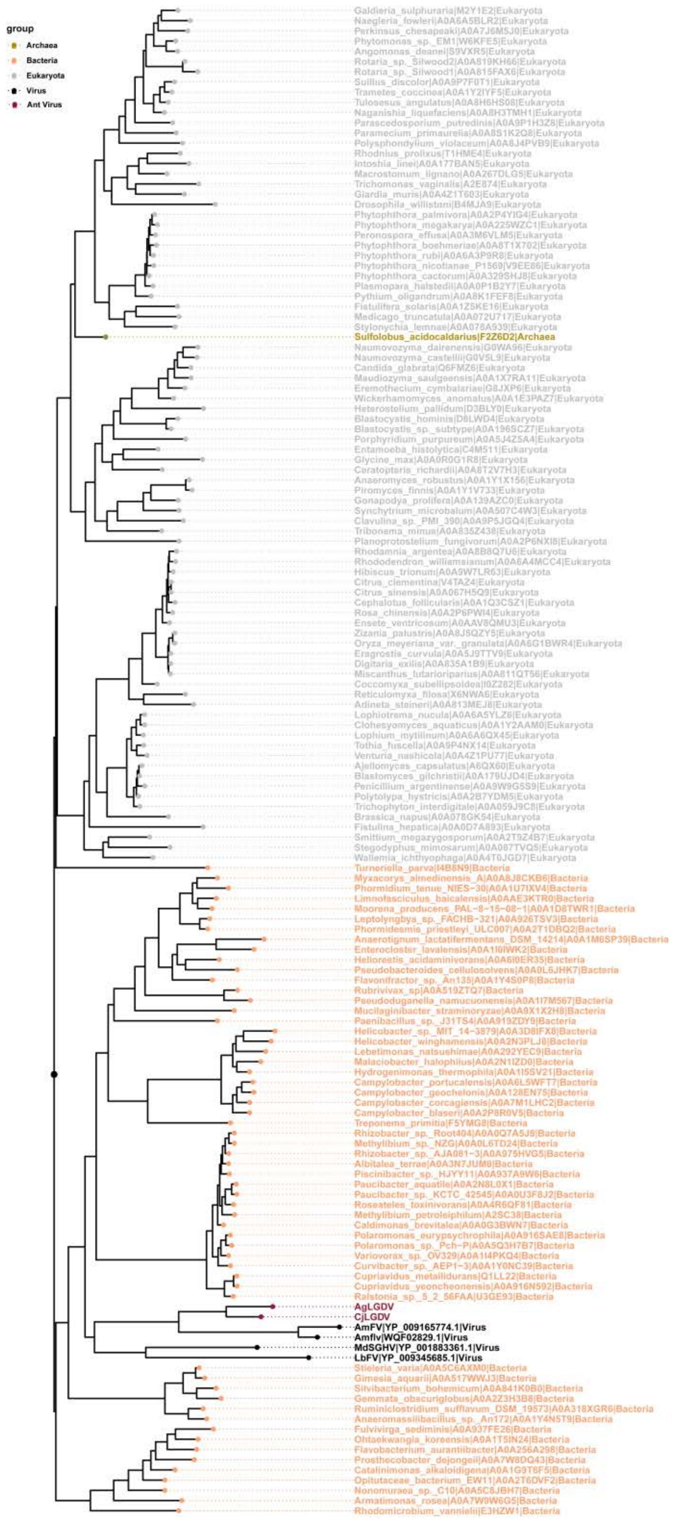

Figure S5C Inhibitors of apoptosis

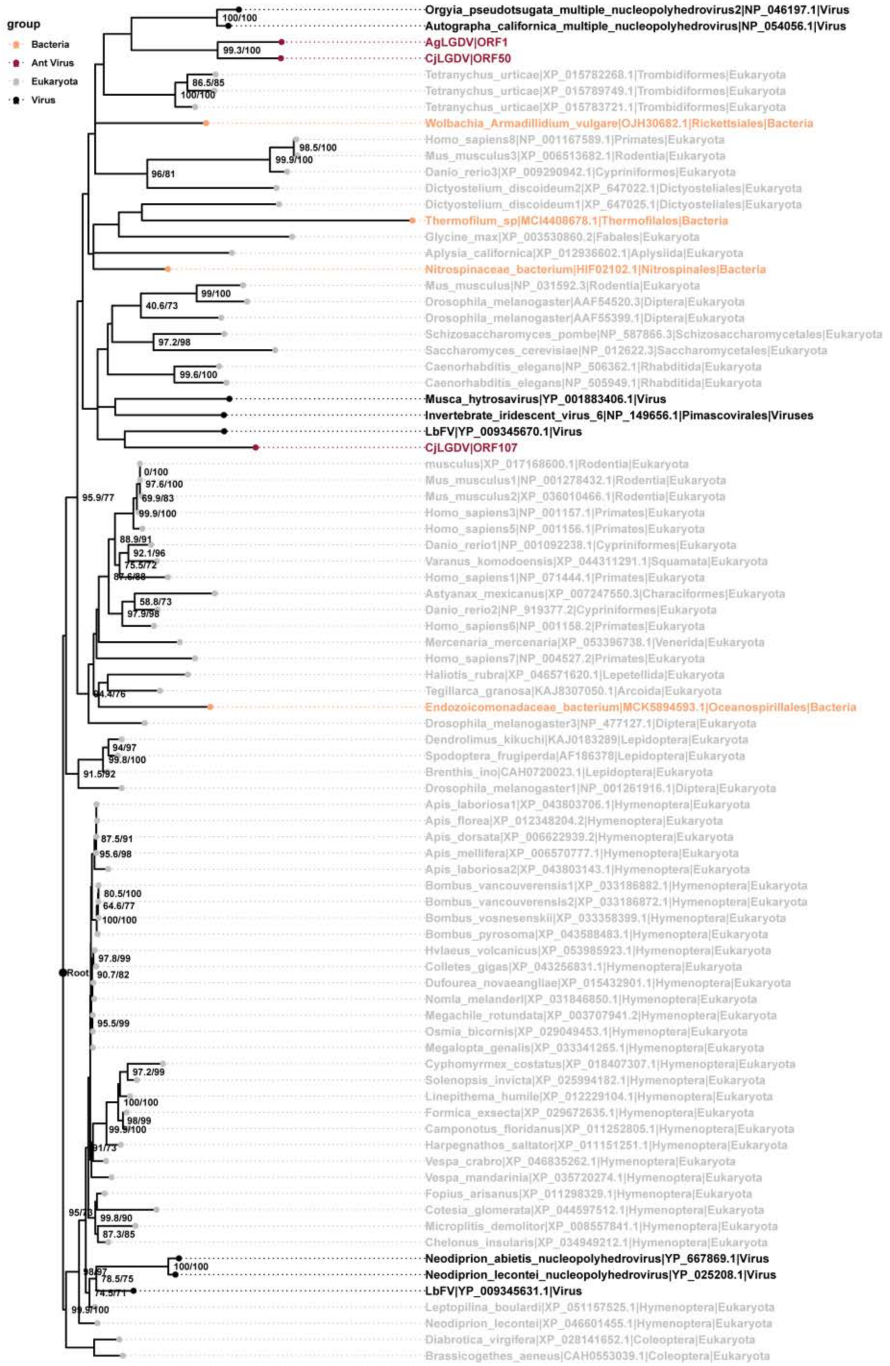

Supplementary Figure S5 Phylogenetic analysis of methyltransferase (A), ATPase (B) and inhibitors of apoptosis (C) genes. The label colors indicate source of the genes: red for CjLGDV and AgLGDV, black for other viruses, grey for eukaryotes and orange for bacteria. Support values are indicated as SH-aLRT support (%) / Ultrafast bootstrap (%).

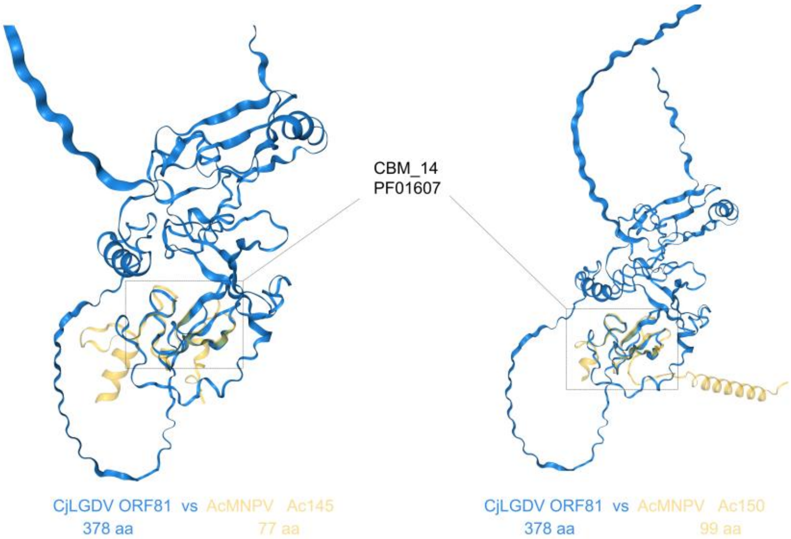

Supplementary Figure S6 Structural alignment of chitin-binding proteins from CjLGDV (ORF81) and AcMNPV (Ac145 and Ac150) by Foldseek. Proteins from different sources are color-coded: blue for CjLGDV, and yellow for AcMNPV.

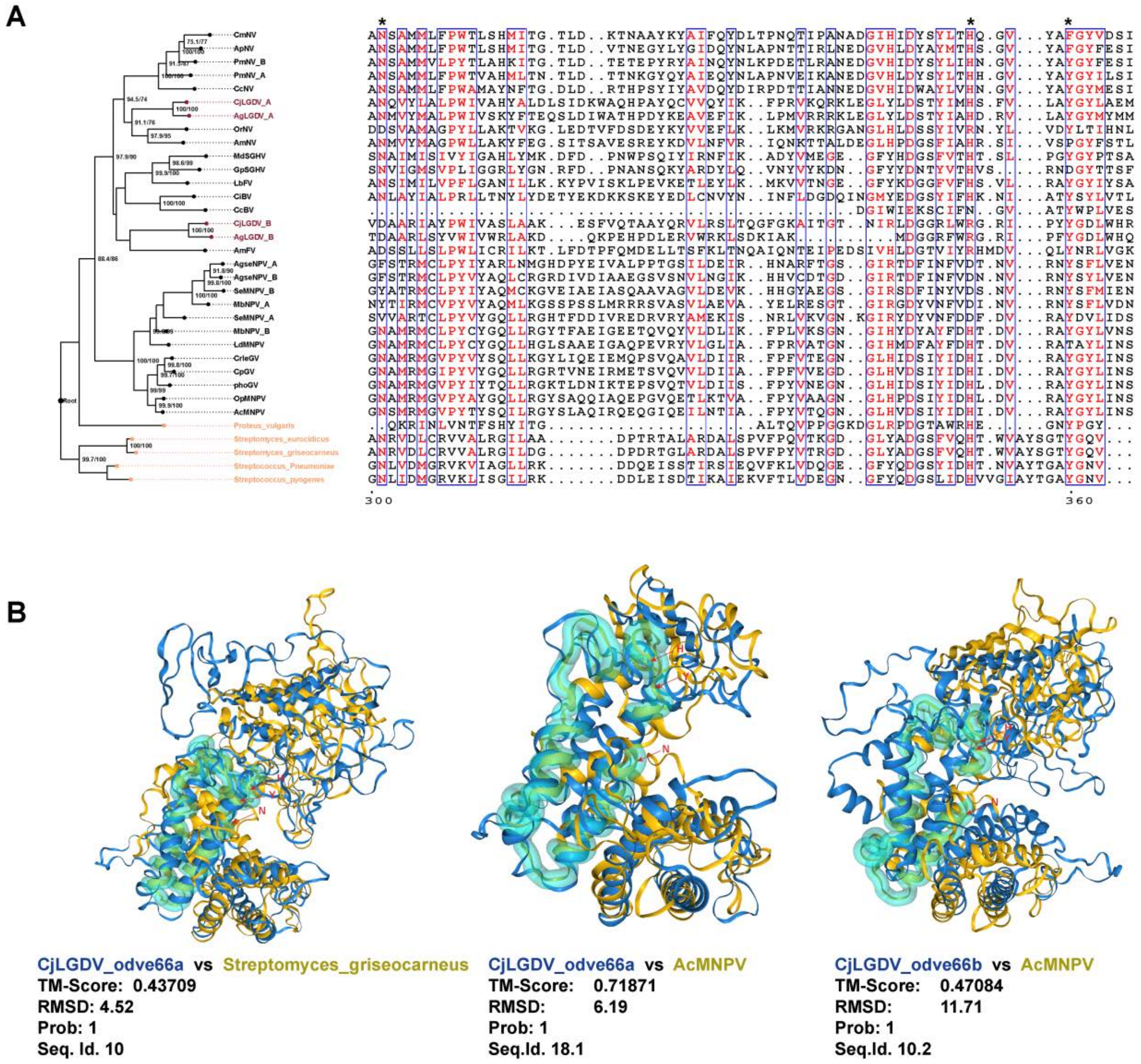

Supplementary Figure S7 (A) Phylogenetic analysis of *odv-e66*. The label colors indicate source of the genes: red for CjLGDV and AgLGDV, black for other viruses and orange for bacteria. Support values are indicated as SH-aLRT support (%) / Ultrafast bootstrap (%). To the right of the phylogenetic tree, a multiple sequence alignment of the region containing the enzyme activity center is shown. The three conserved residues (N, H, and Y) crucial for enzyme activity are highlighted with asterisks. (B) Protein structure alignments of CjLGDV ODV-E66 homologs with the proteins derived from a bacterium and AcMNPV by Foldseek. Different colors indicate the source of the genes: blue for CjLGDV, yellow for AcMNPV or the indicated bacterium. The enzyme active center is highlighted in green. The three conserved amino acids are indicated with red arrows.

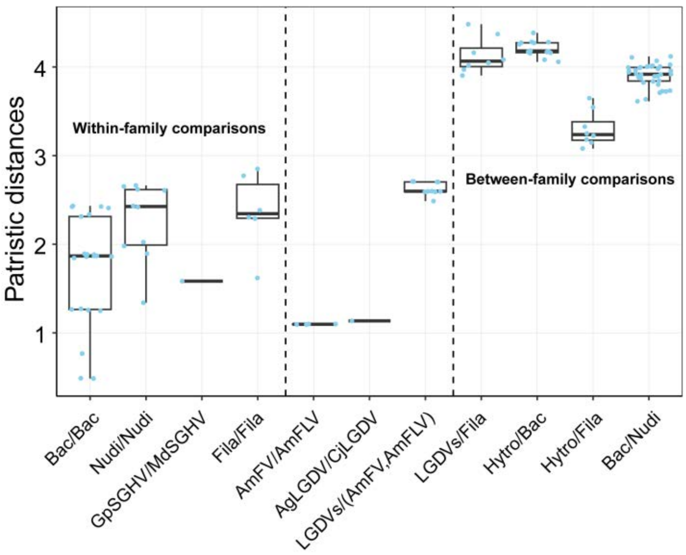

Supplementary Figure S8 Patristic distances within and between virus families in *Lefavirales*. Patristic distances were calculated based on the phylogenetic tree shown in Fig.4.

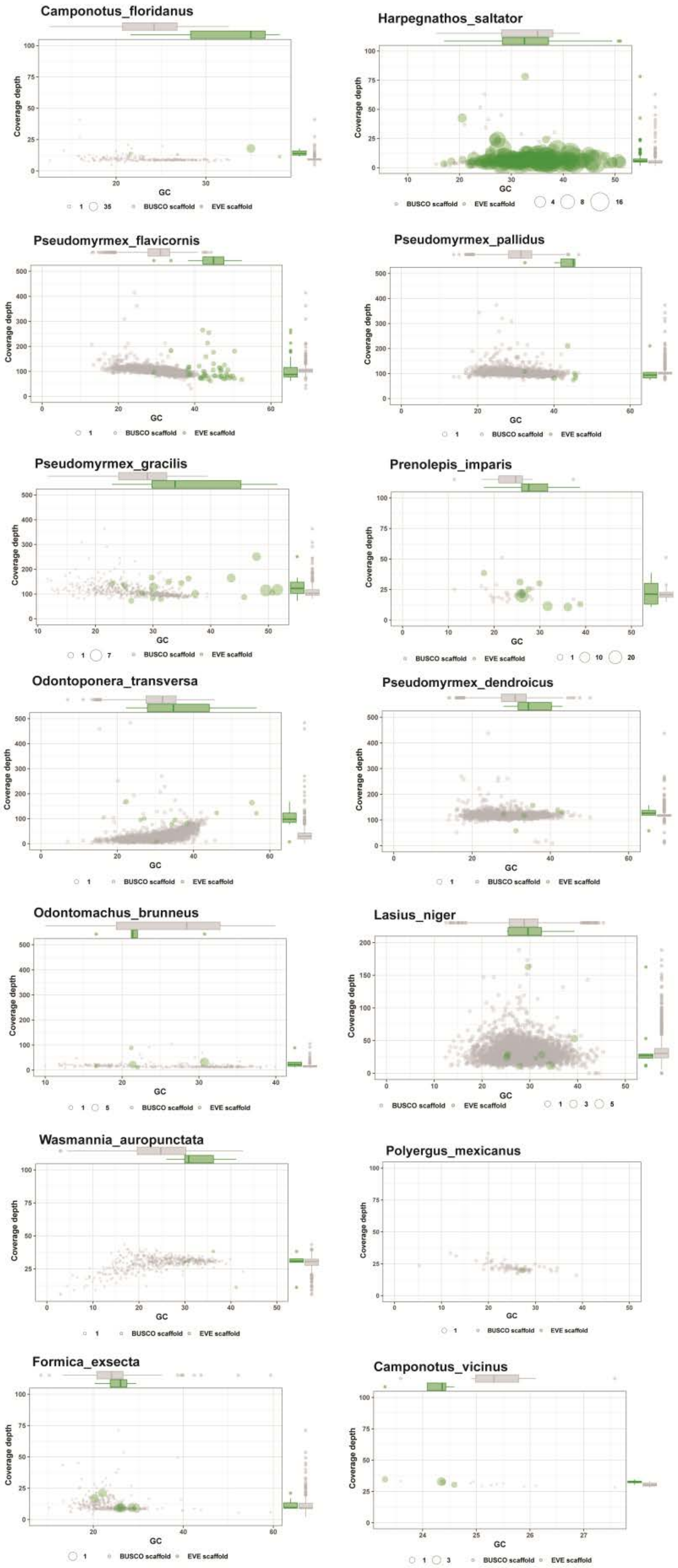
Camponotus_floridanus
Harpegnathos_saltator
Pseudomyrmex_flavicornis
Pseudomyrmex_pallidus
Pseudomyrmex_gracilis
Prenolepis_imparis
Odontoponera_transversa
Pseudomyrmex_dendroicus
Odontomachus_brunneus
Lasius_niger
Wasmannia_auropunctata
Polyergus_mexicanus
Formica_exsecta
Camponotus_vicinus
Coverage depth
GC
BUSCO scaffold
EVE scaffold

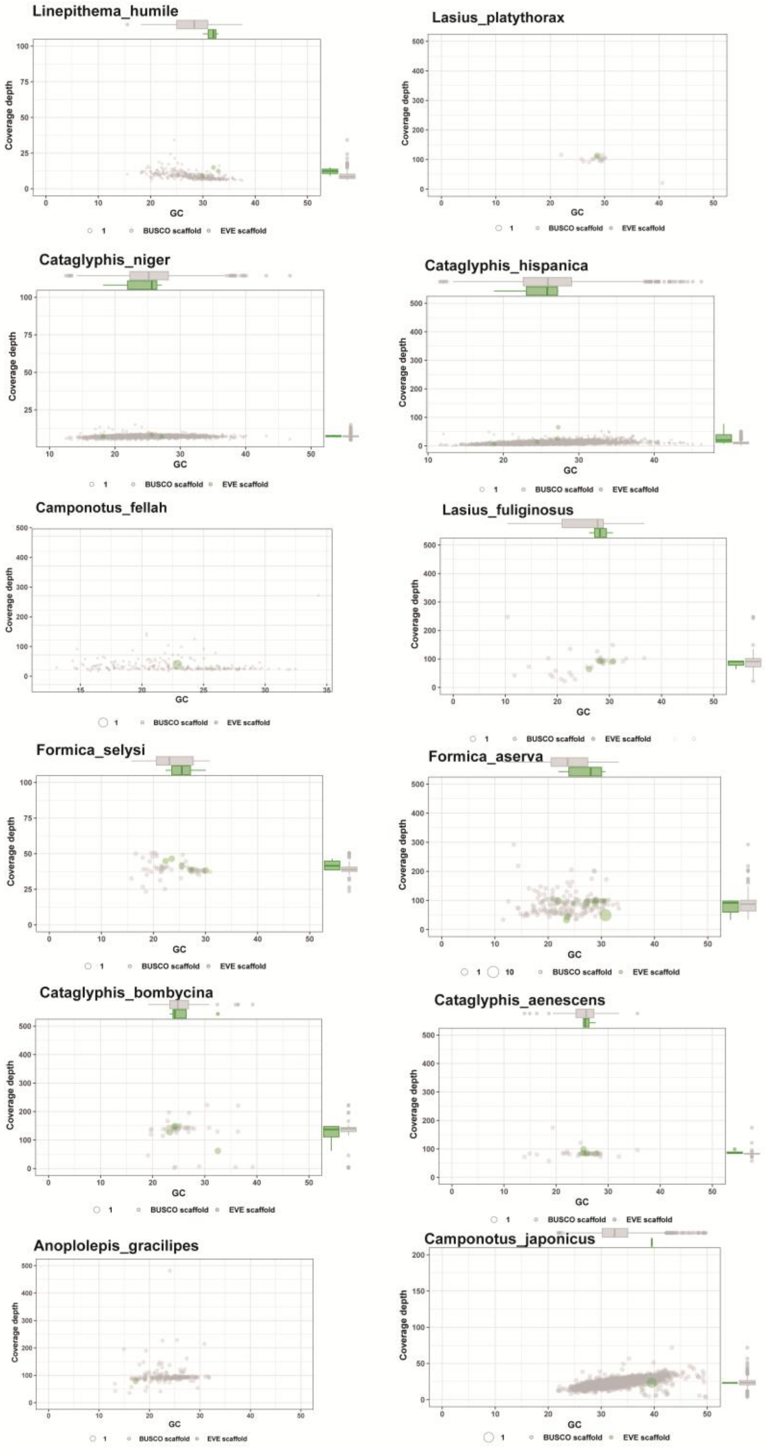

Supplementary Figure S9 GC content and coverage depth of scaffolds containing endogenous viral elements (EVEs). Each dot represents a scaffold, with the x-axis showing GC content and the y-axis showing sequencing depth. Green dots indicate scaffolds containing EVEs. Grey dots represent scaffolds with BUSCO genes. Dot size reflects the number of candidate EVEs per scaffold.

Figure S10-1 DNApol

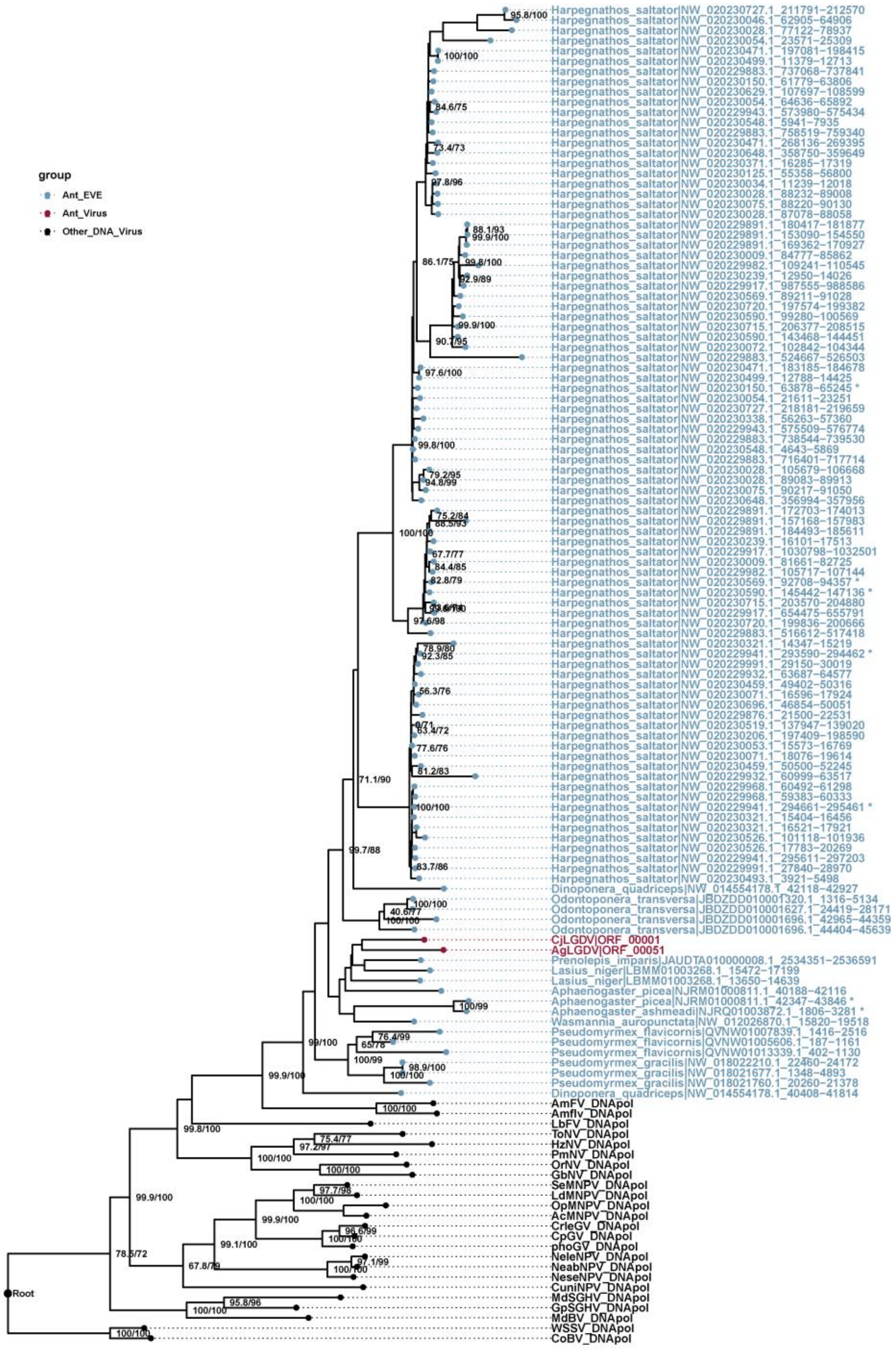

Figure S10-2 CjLGDV_ORF7

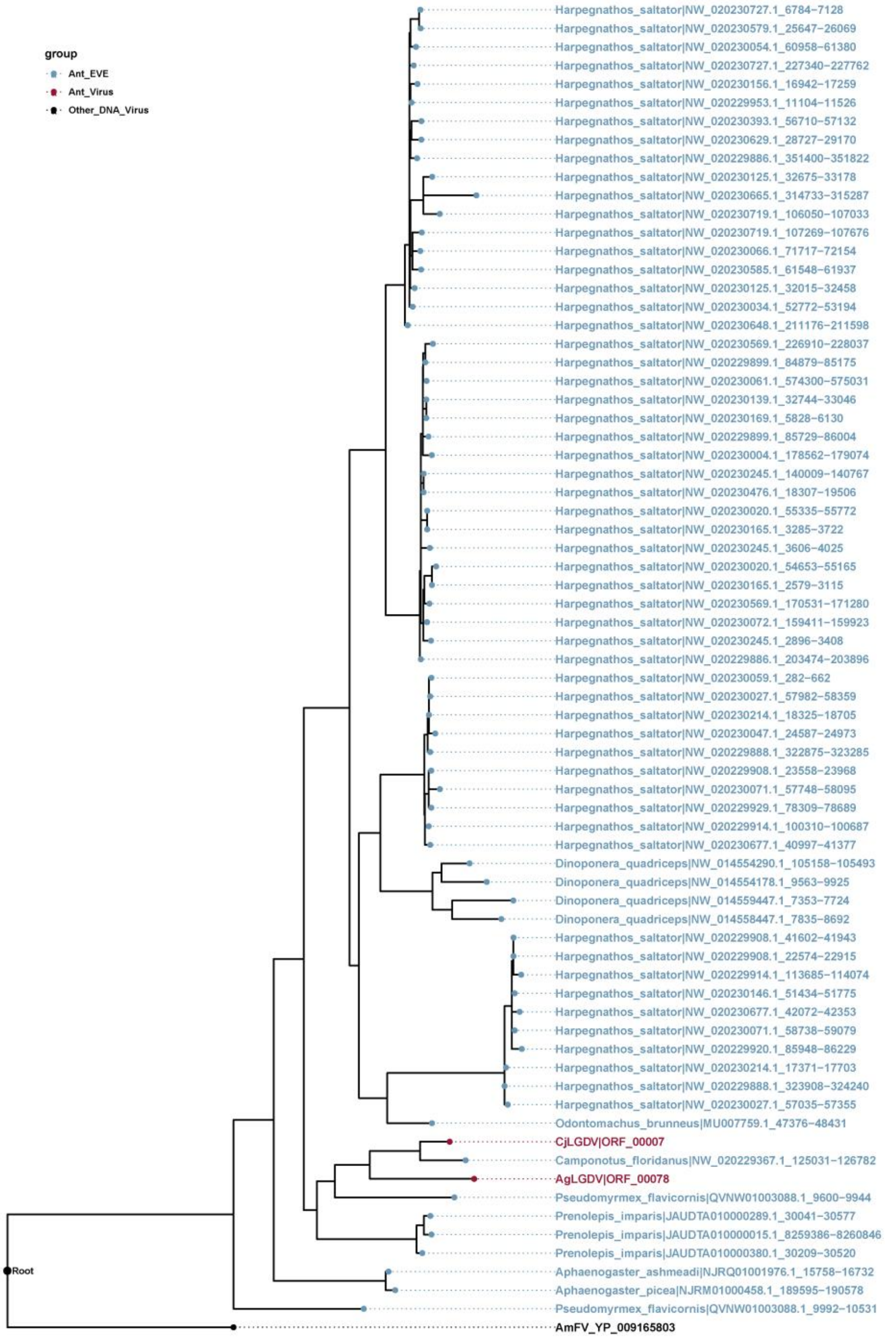

Figure S10-3 ODV-E66 CjLGDV_ORF9/11

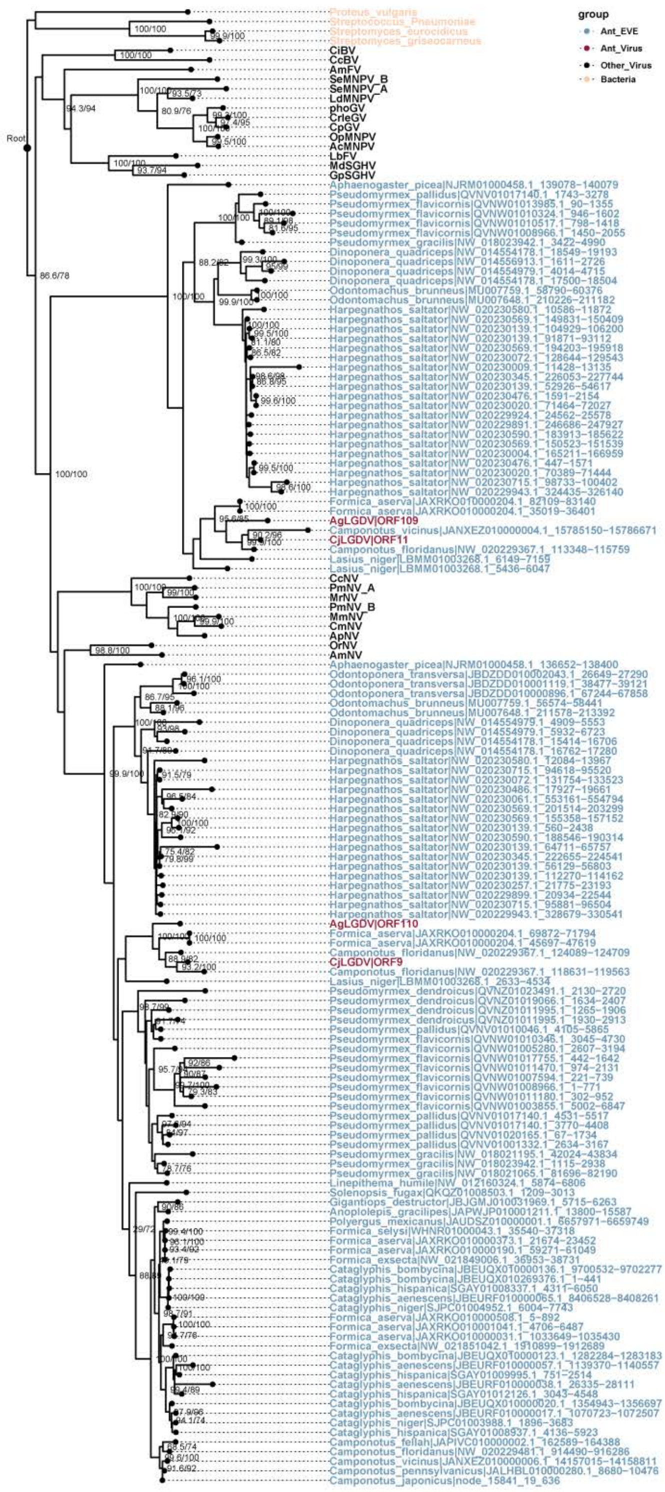

Figure S10-4 Helicase2 CjLGDV_ORF20

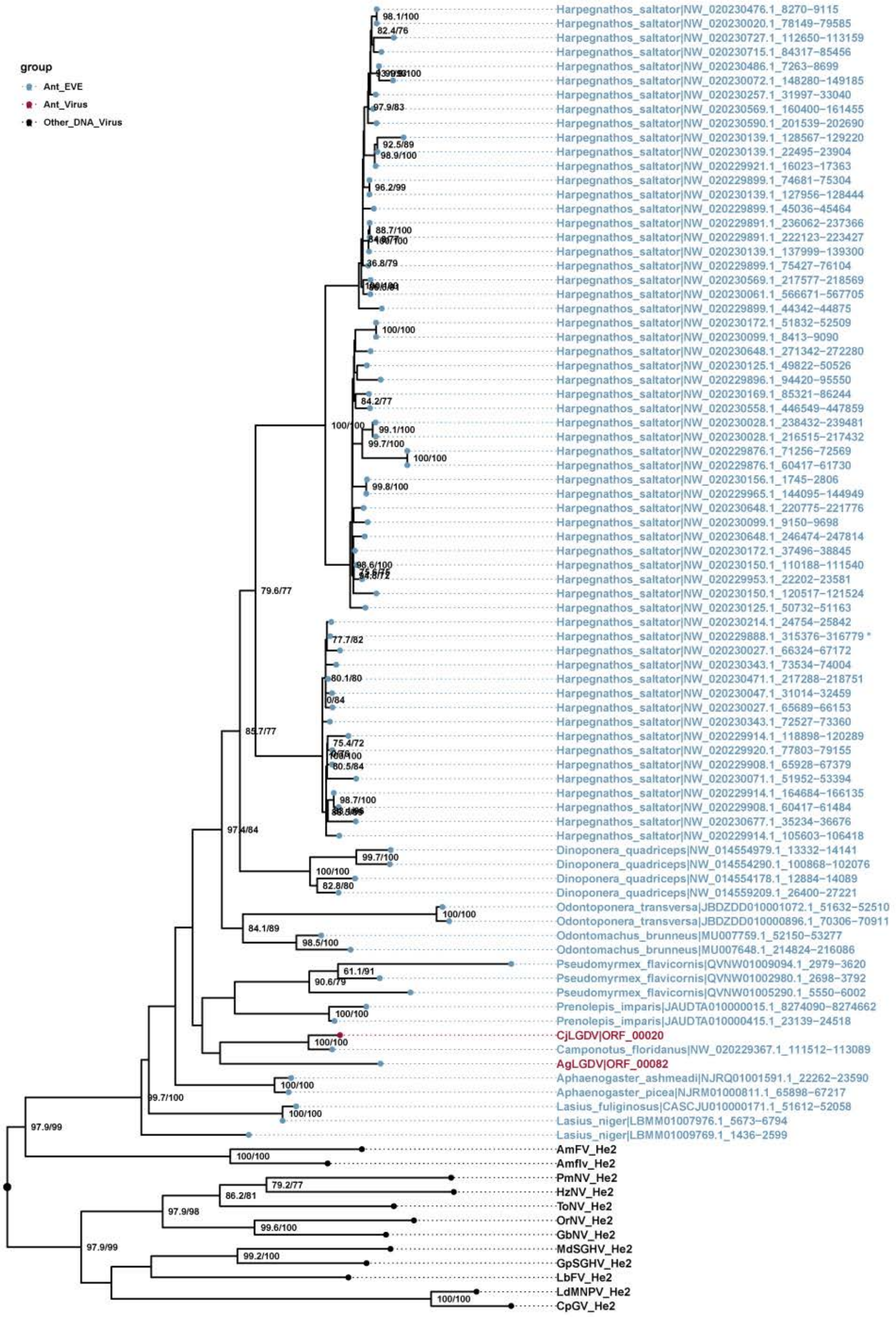

Figure S10-5 CjLGDV_ORF23

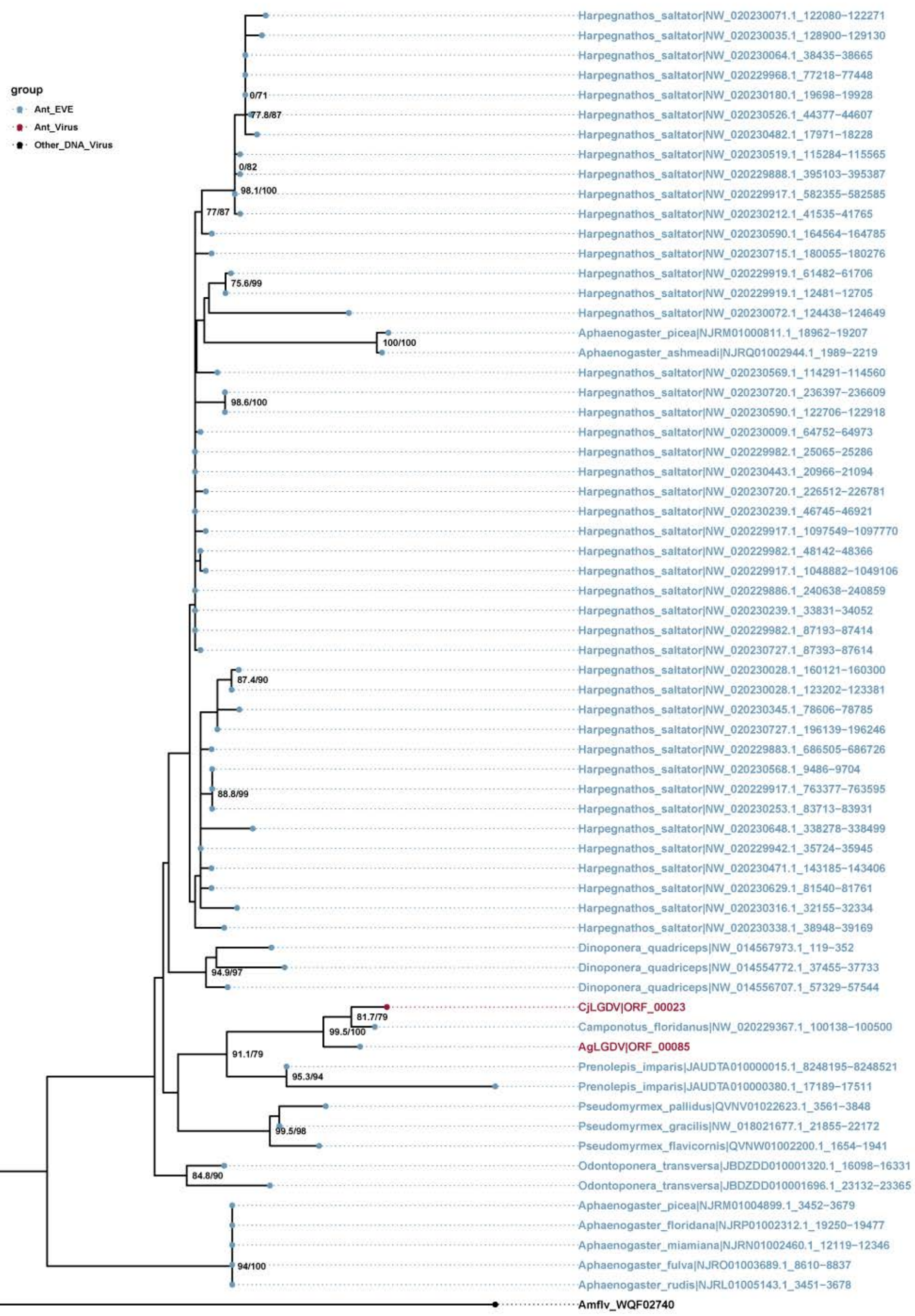

Figure S10-6 PIF4 CjLGDV_ORF24

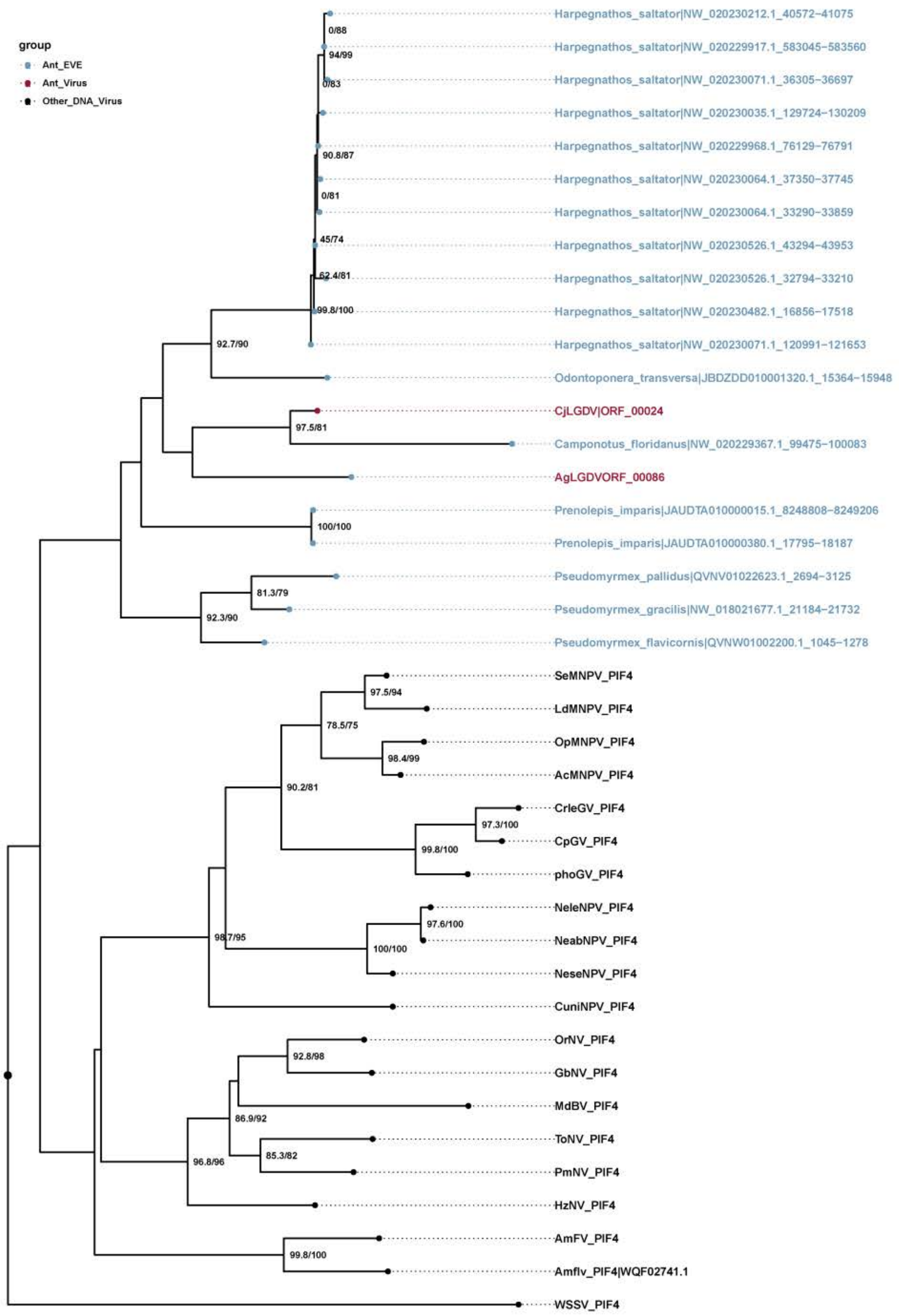

Figure S10-7 CjLGDV_ORF25

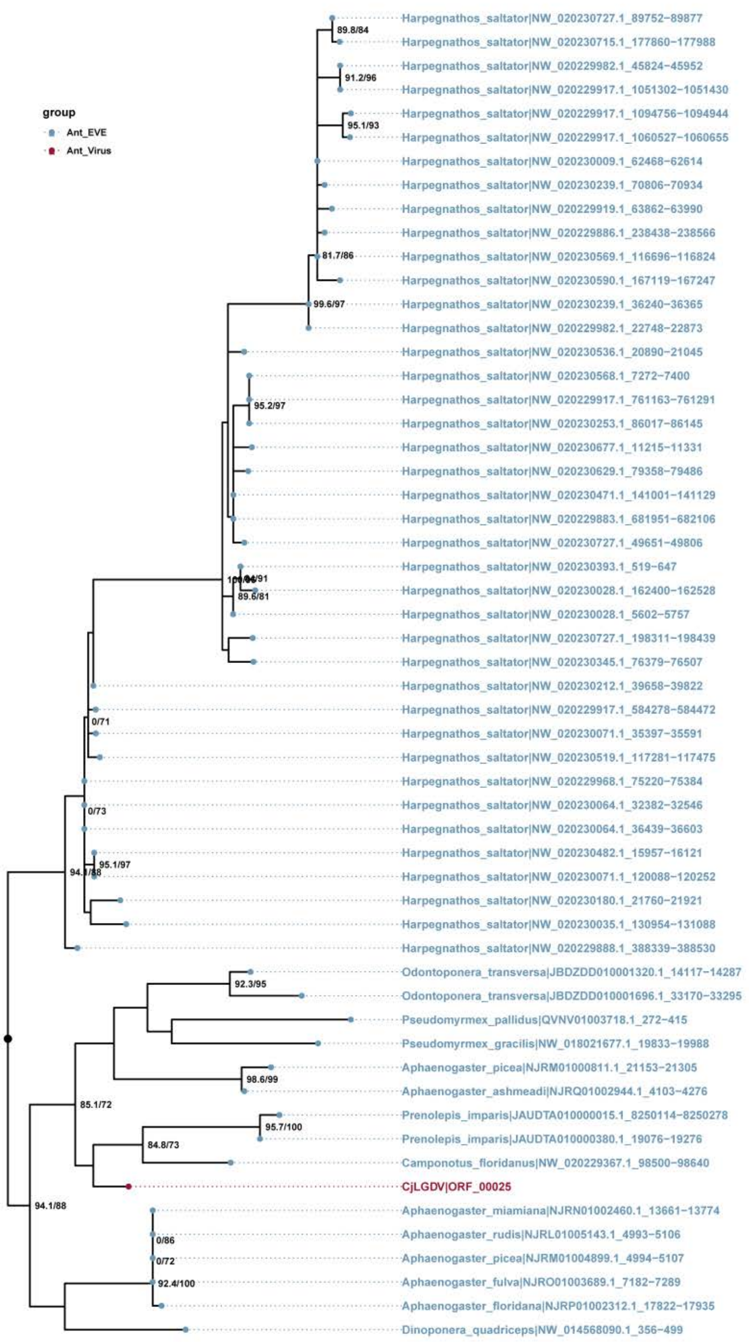

Figure S10-8 LEF4 CjLGDV_ORF29

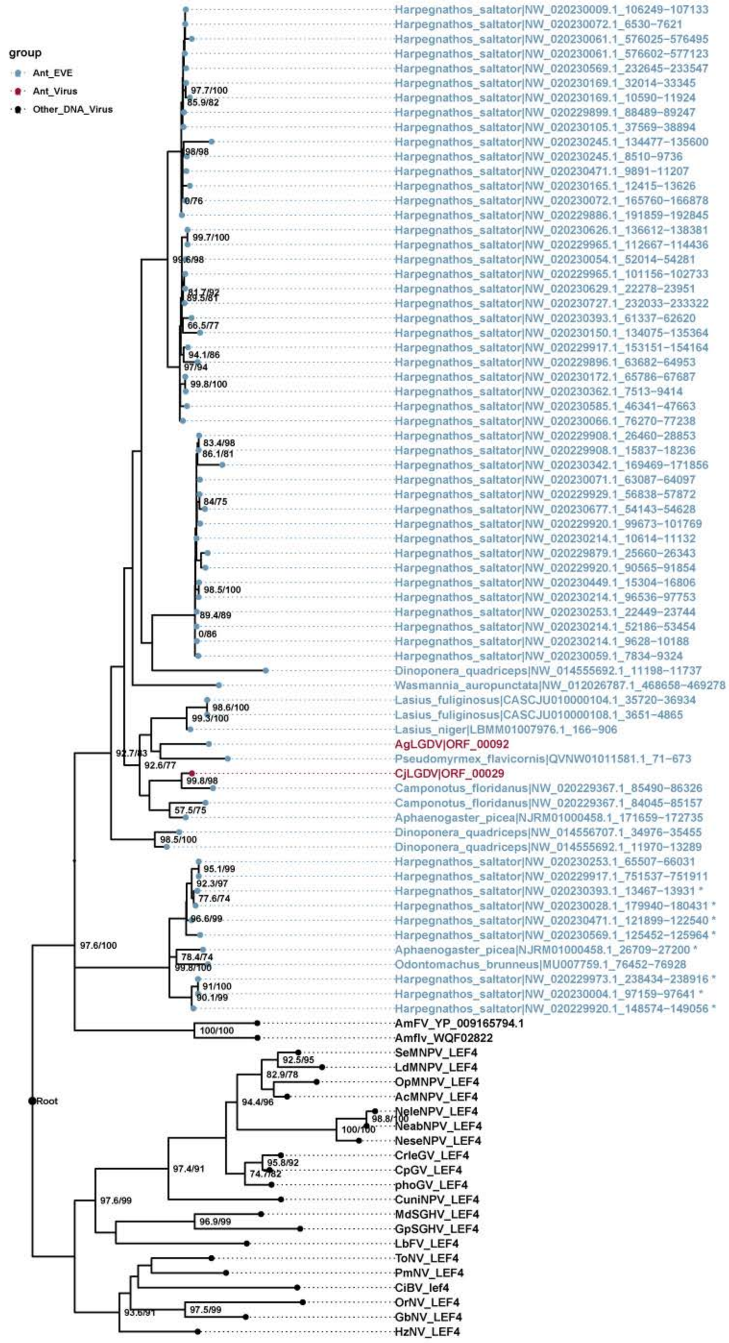

Figure S10-9 Integrase CjLGDV_ORF32

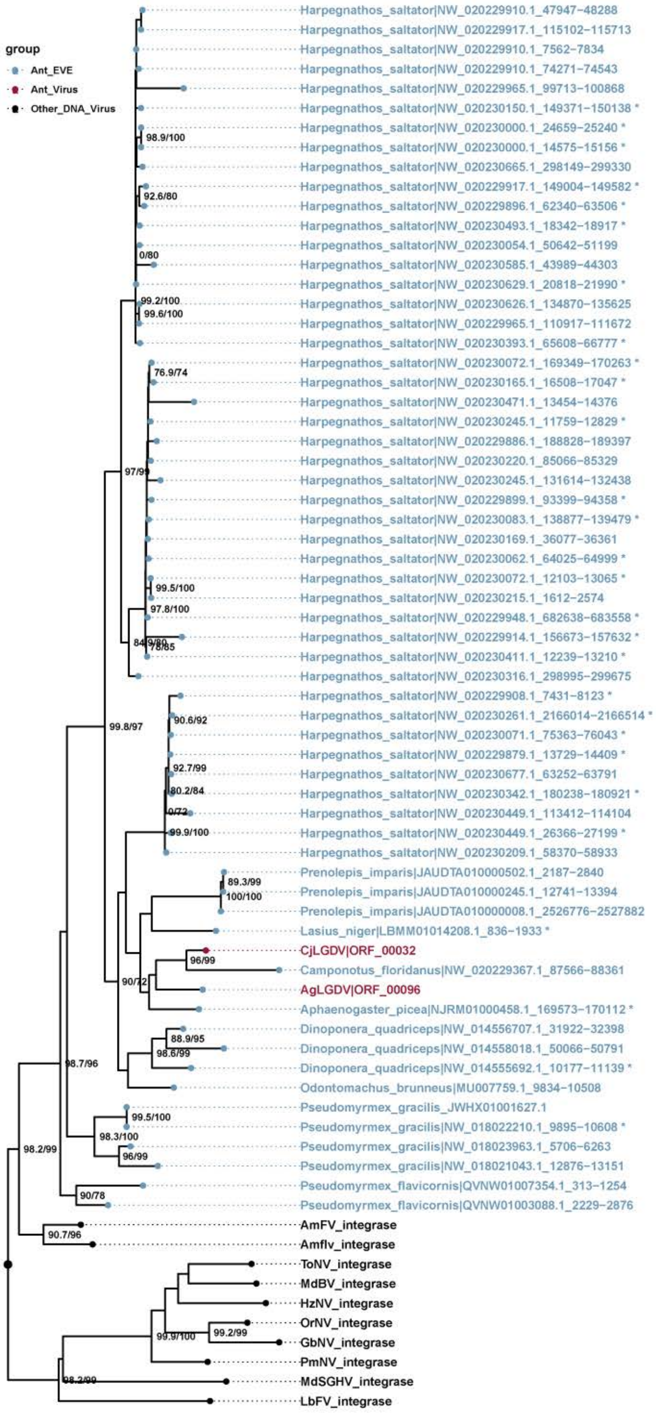

Figure S10-10 LEF9 CjLGDV_ORF34

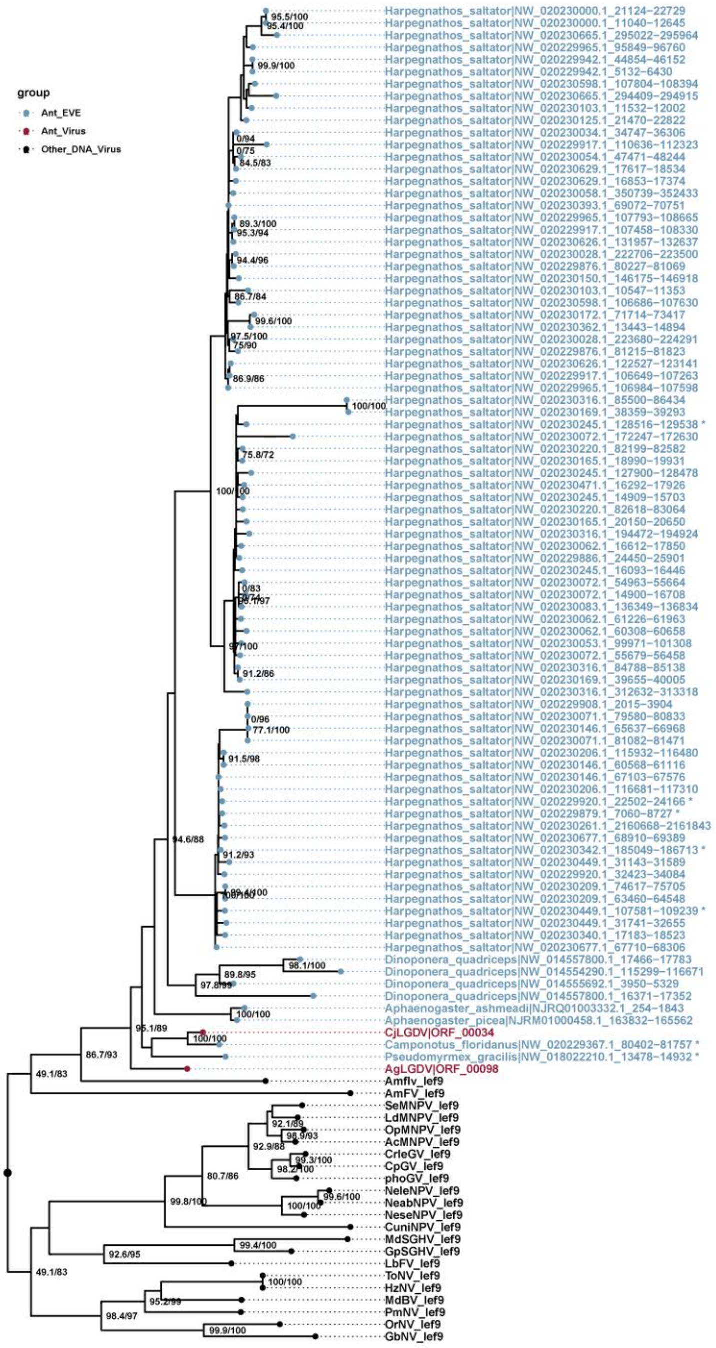

Figure S10-11 CjLGDV_ORF41

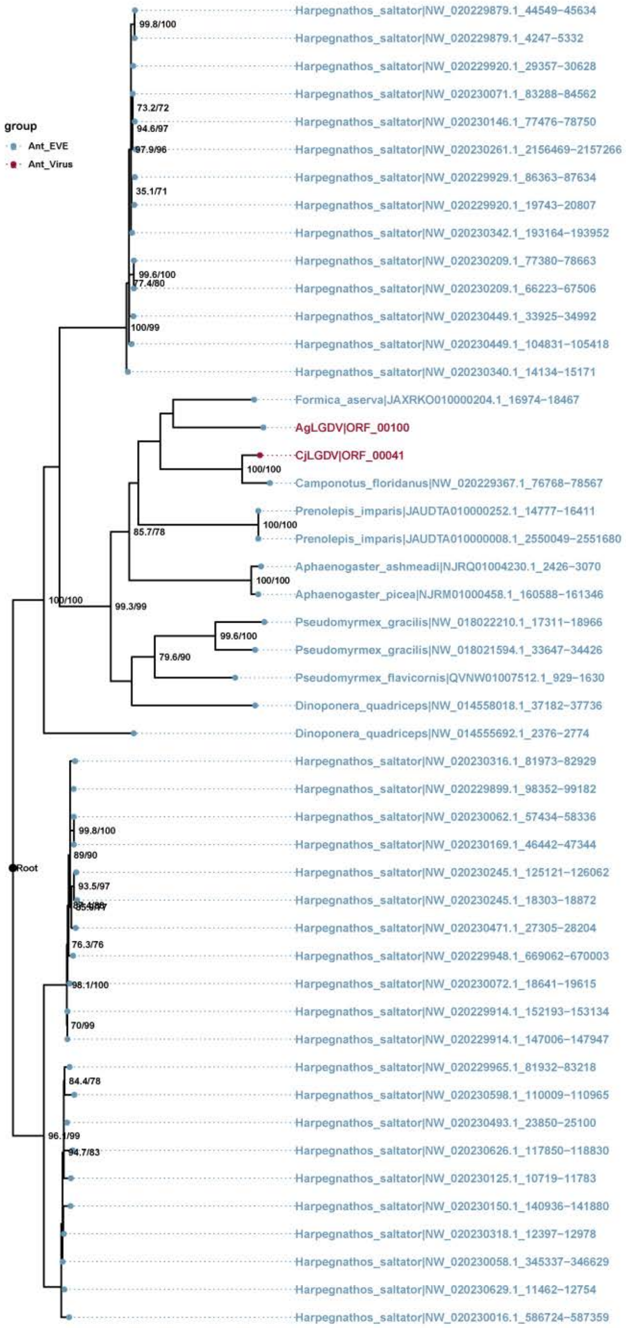

Figure S10-12 PIF3 CjLGDV_ORF45

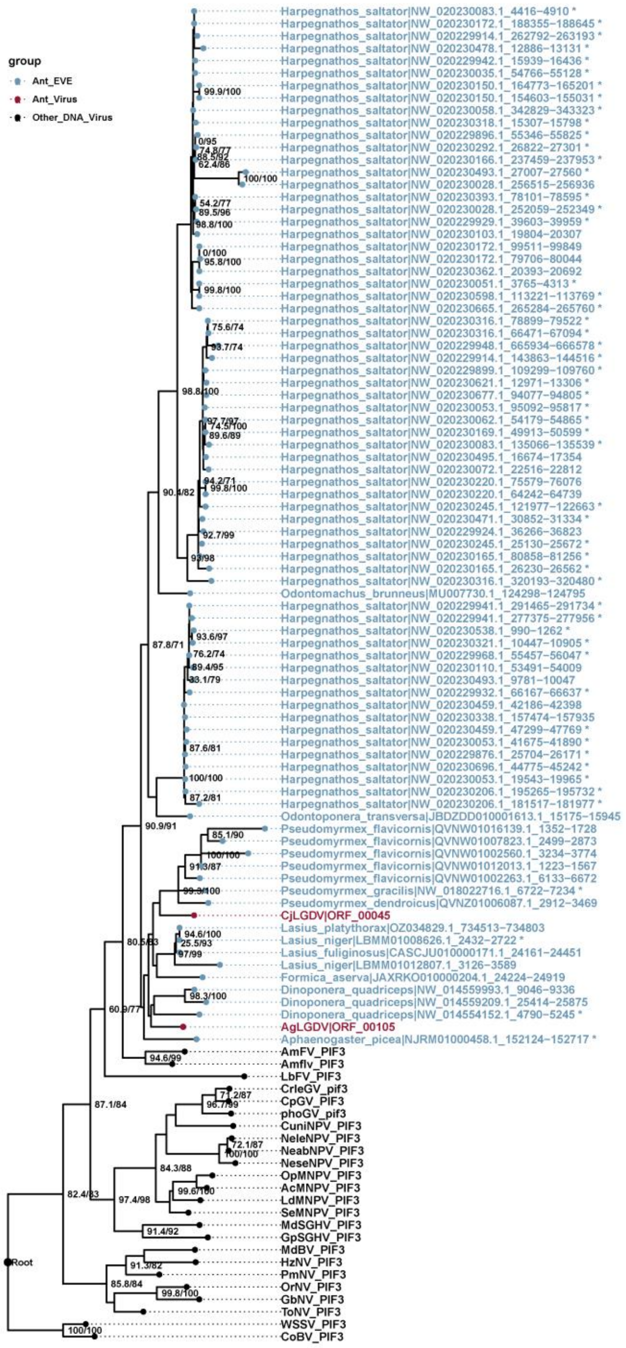

Figure S10-13 CjLGDV_ORF47

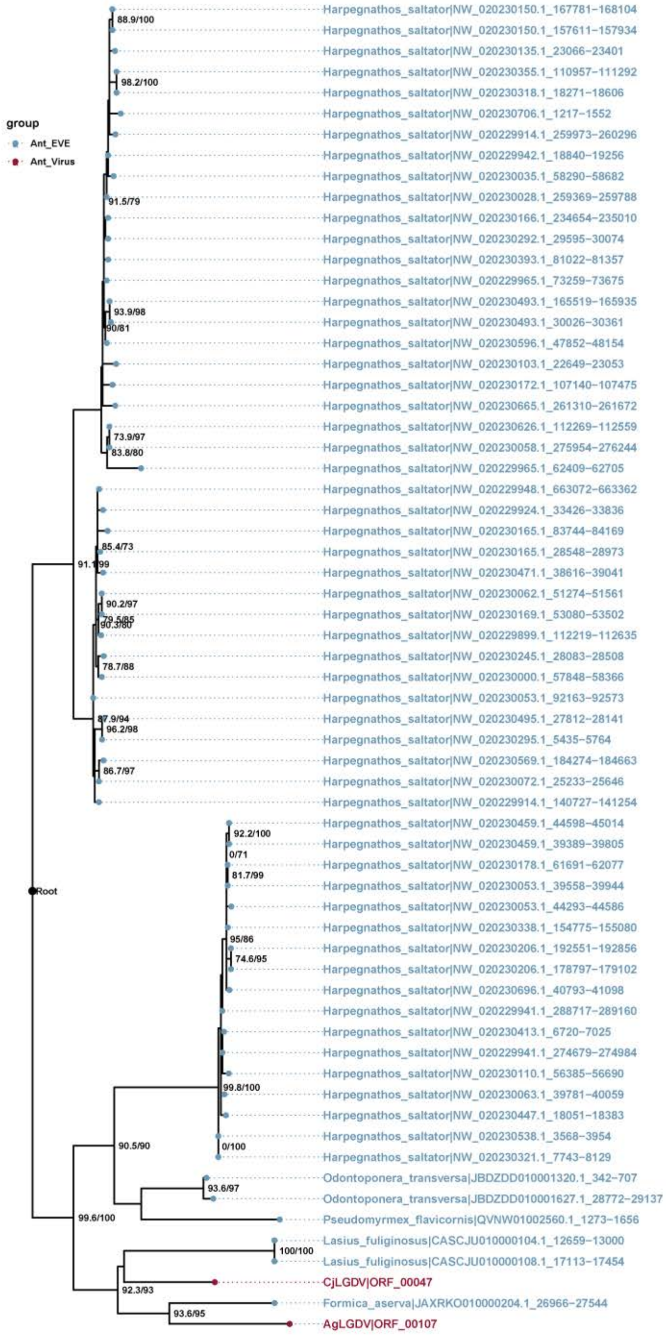

Figure S10-14 CjLGDV_ORF48

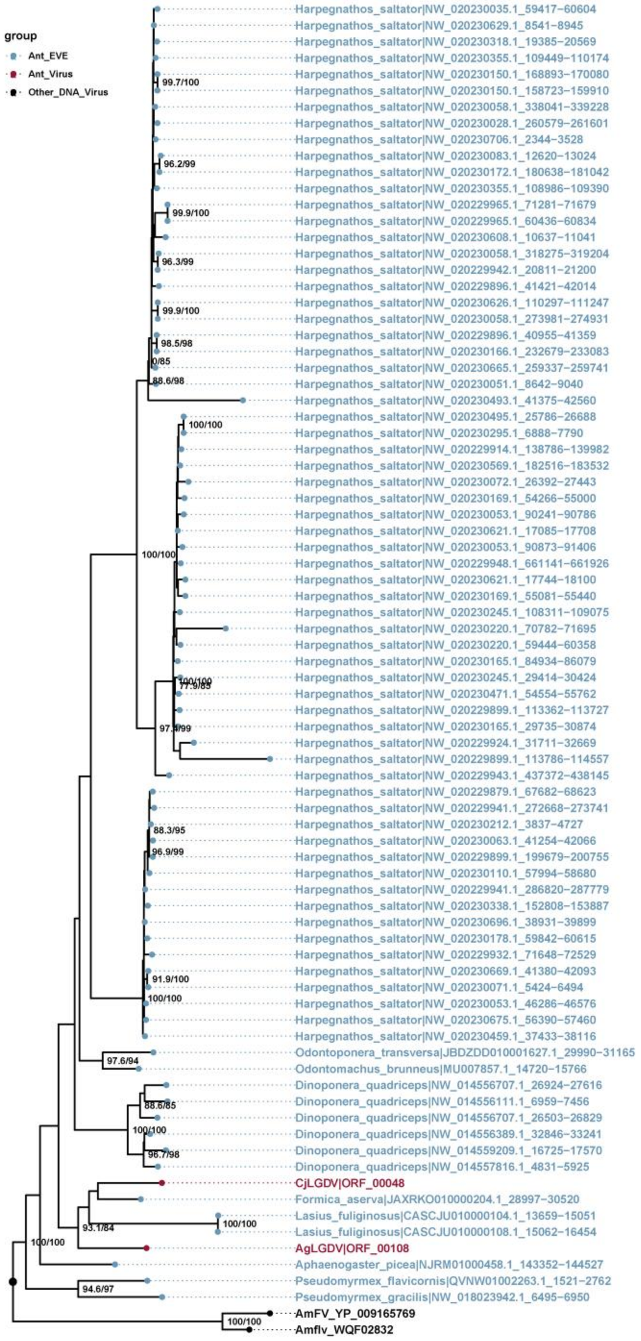

Figure S10-15 Helicase CjLGDV_ORF59

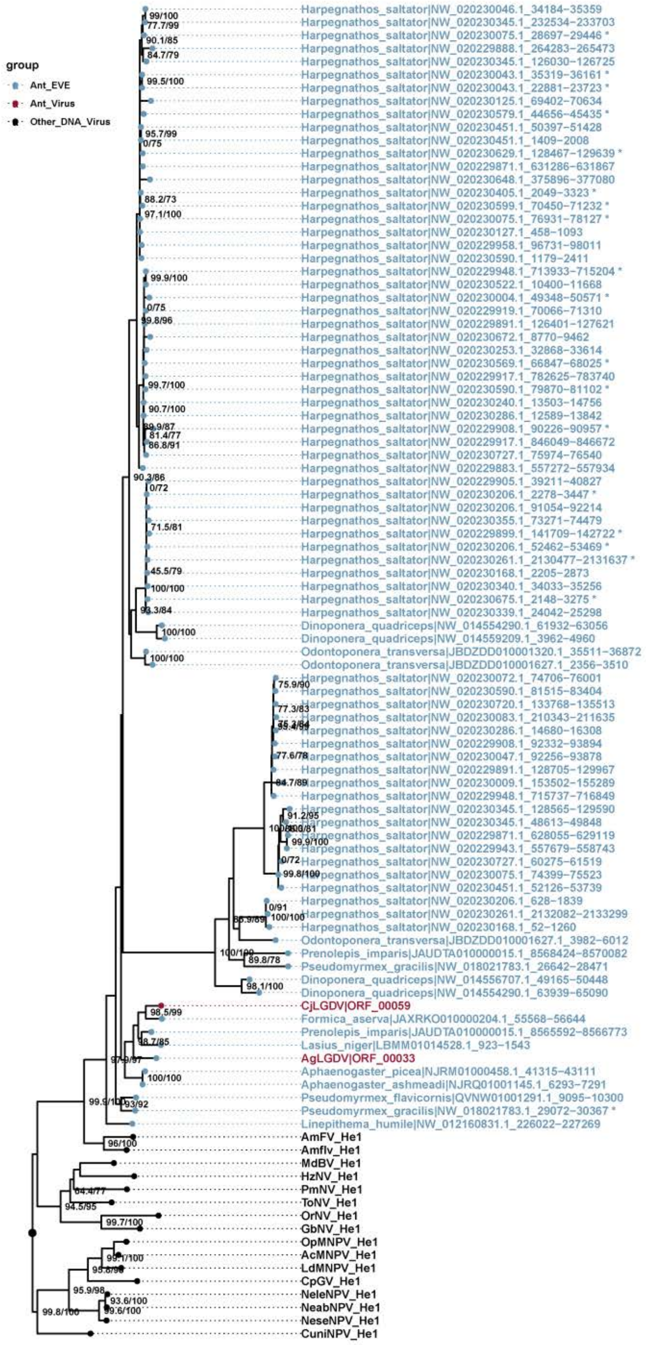

Figure S10-16 Methyltransferase CjLGDV_ORF60

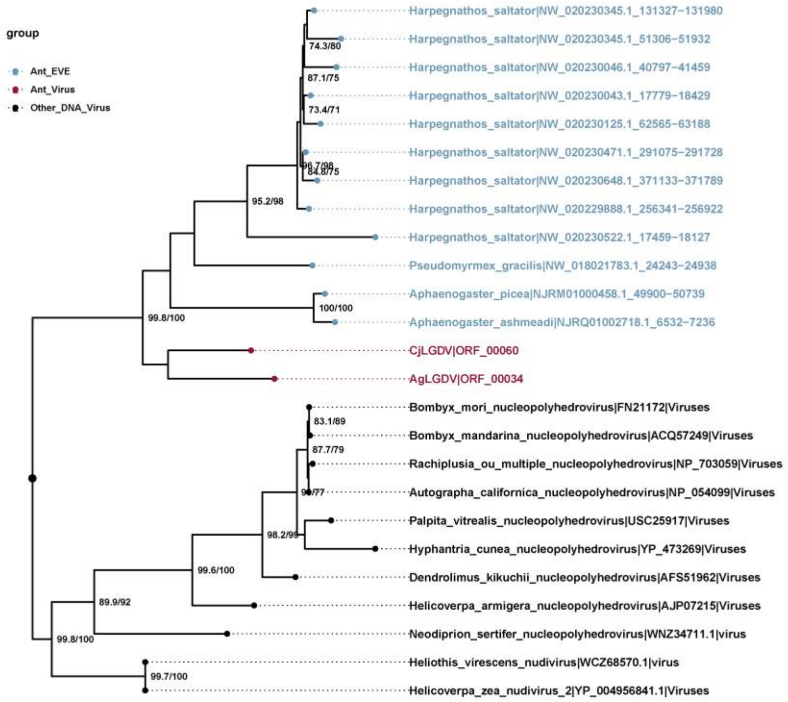

Figure S10-17 PIF2 CjLGDV_ORF64

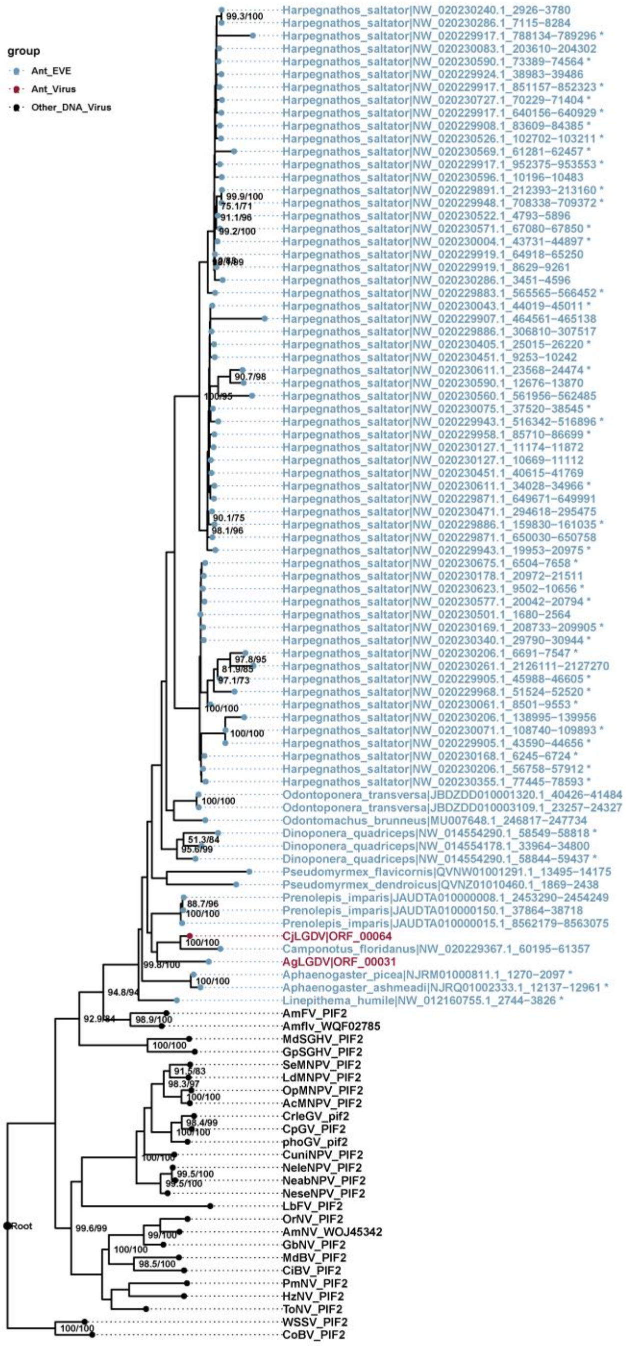

Figure S10-18 P74 CjLGDV_ORF73

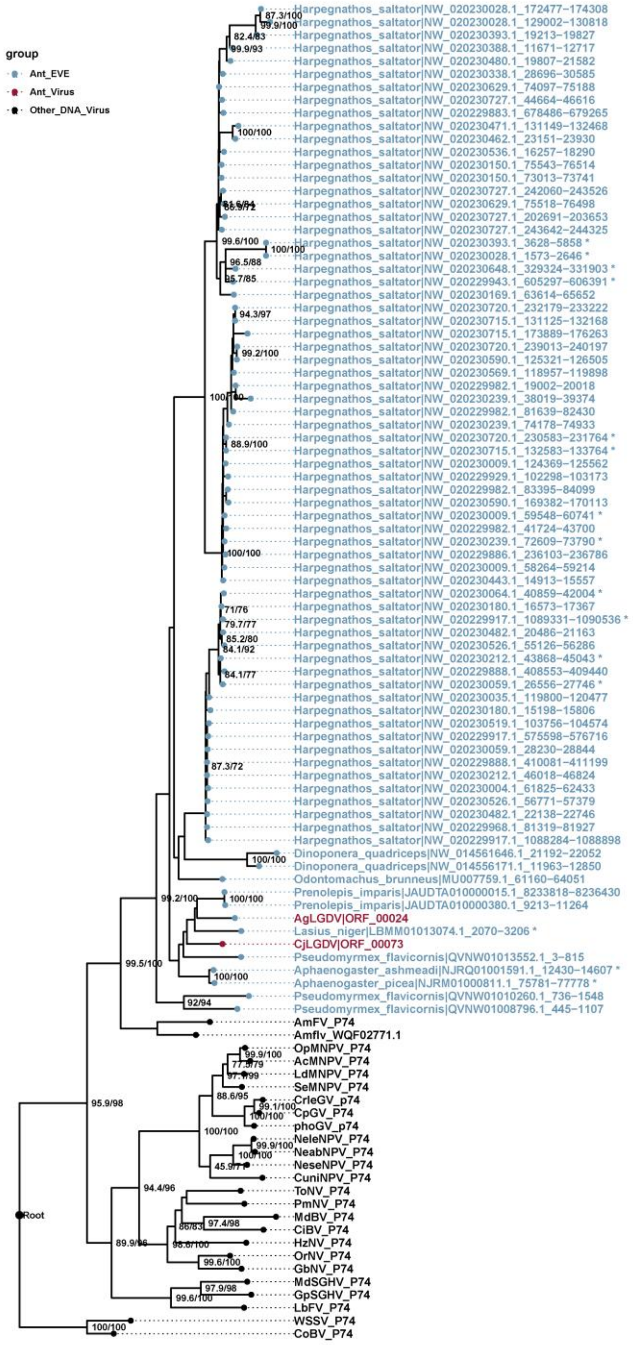

Figure S10-19 CjLGDV_ORF77

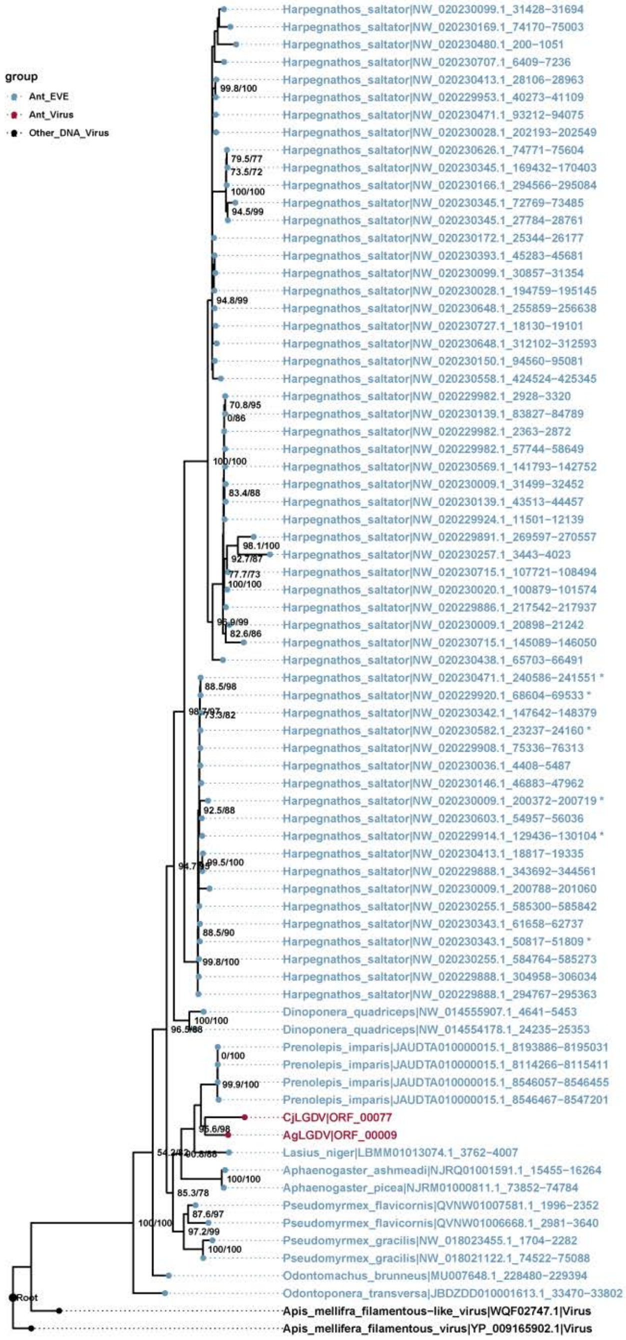

Figure S10-20 LEF5 CjLGDV_ORF83

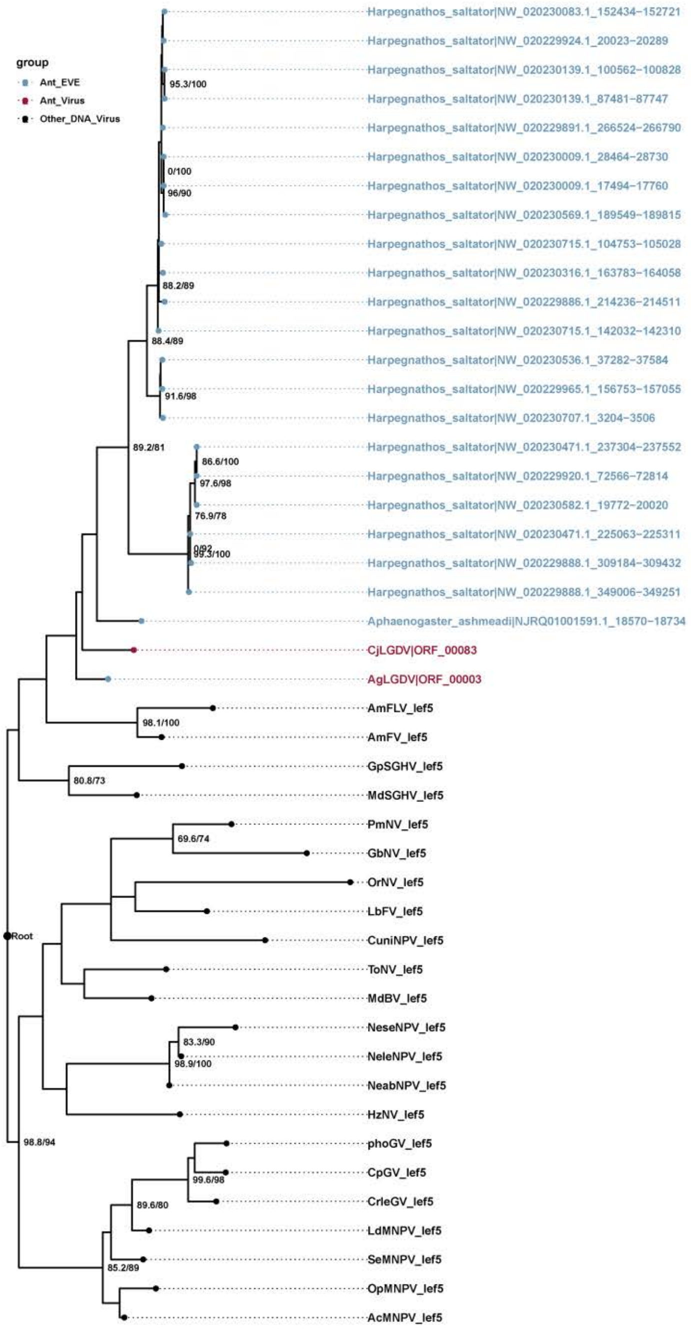

Figure S10-21 P33 CjLGDV_ORF84

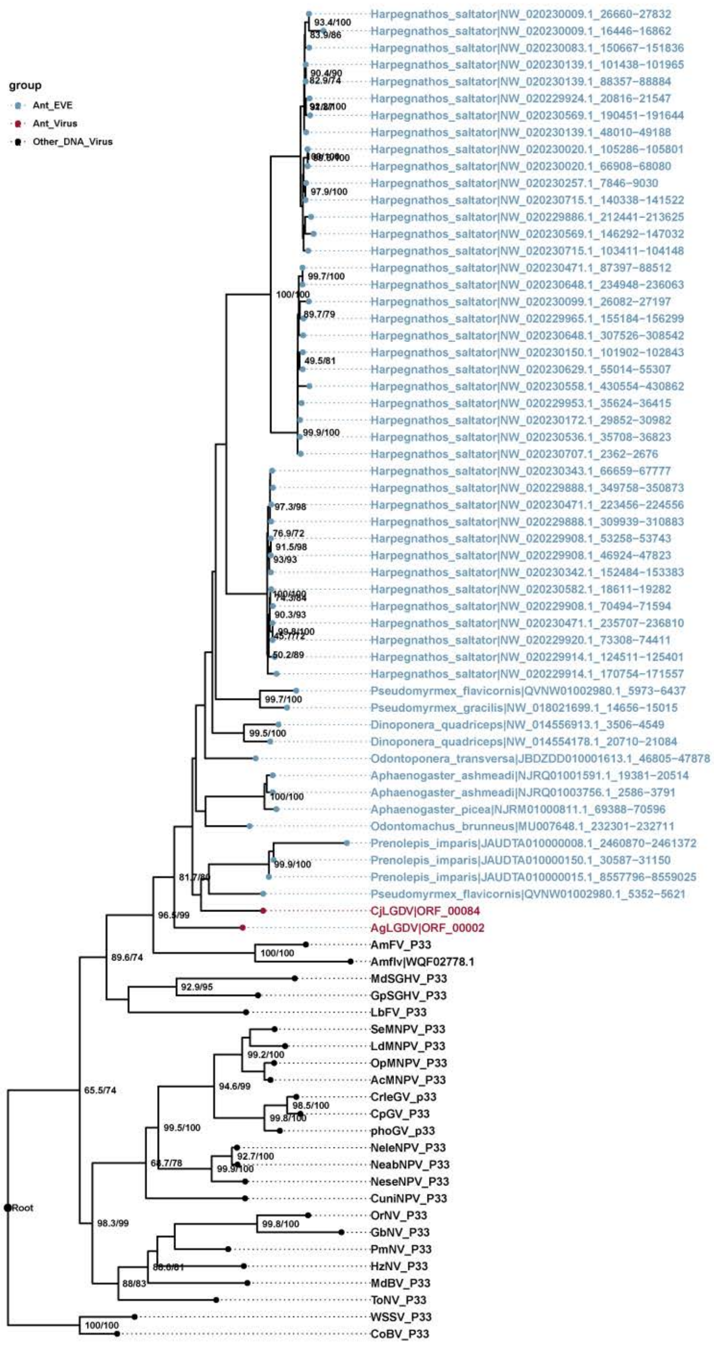

Figure S10-22 CjLGDV_ORF86

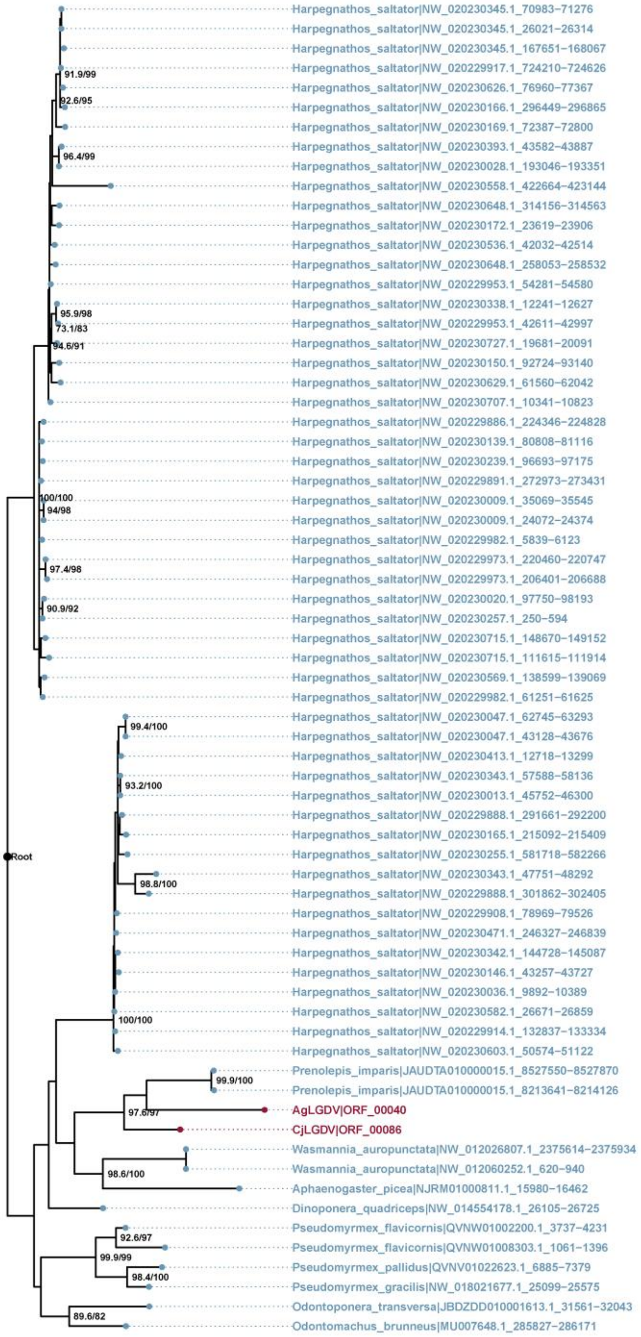

Figure S10-23 PIF5 CjLGDV_ORF87

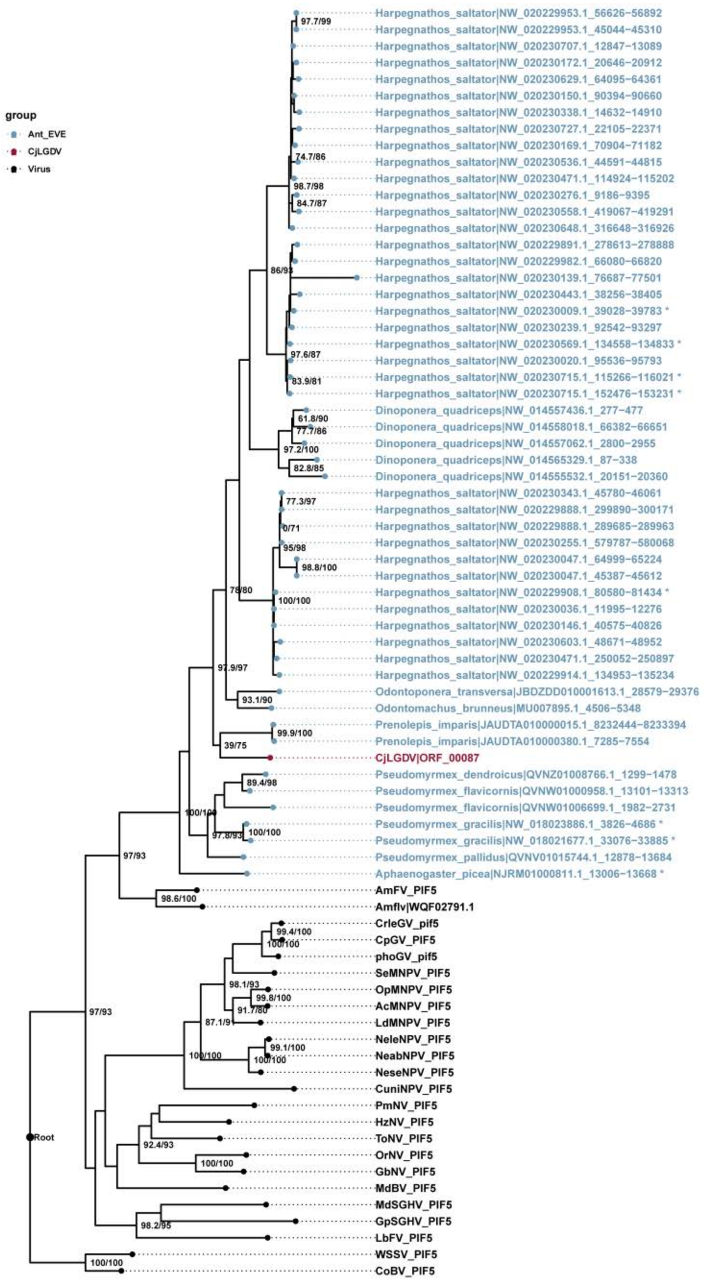

Figure S10-24 CjLGDV_ORF94

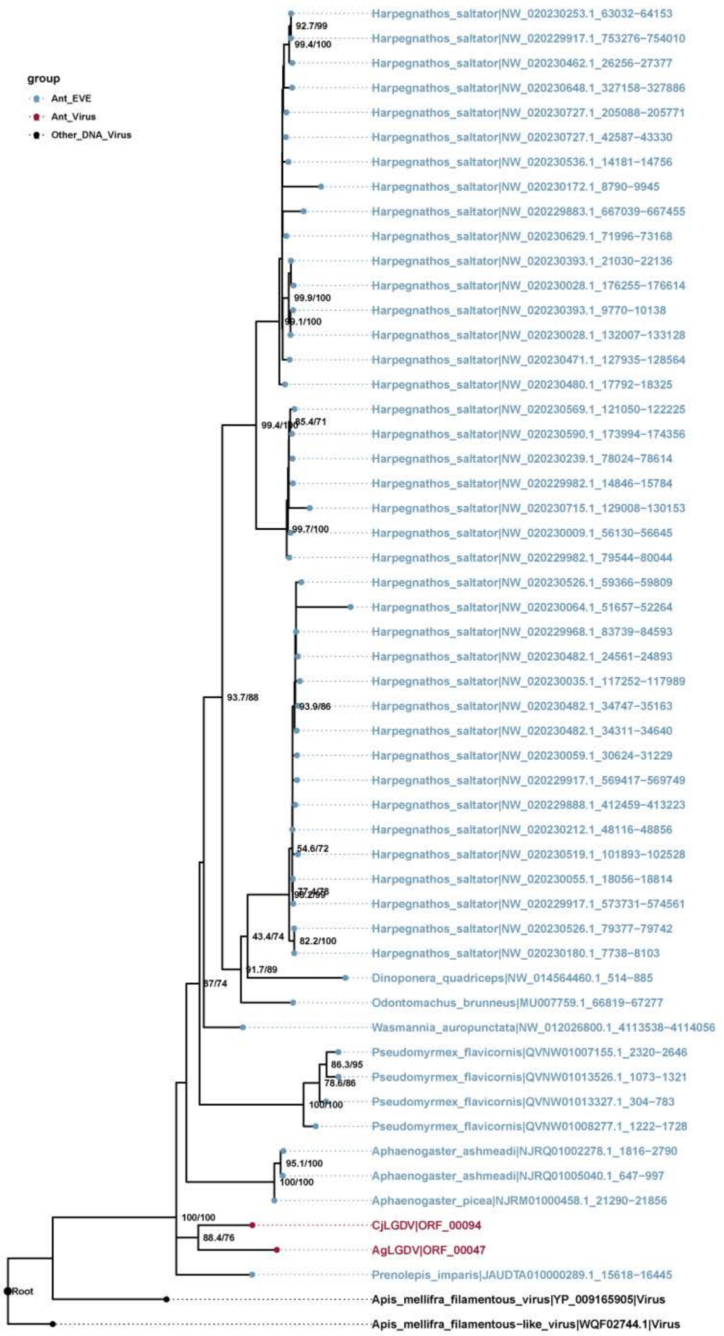

Figure S10-25 LEF8 CjLGDV_ORF95

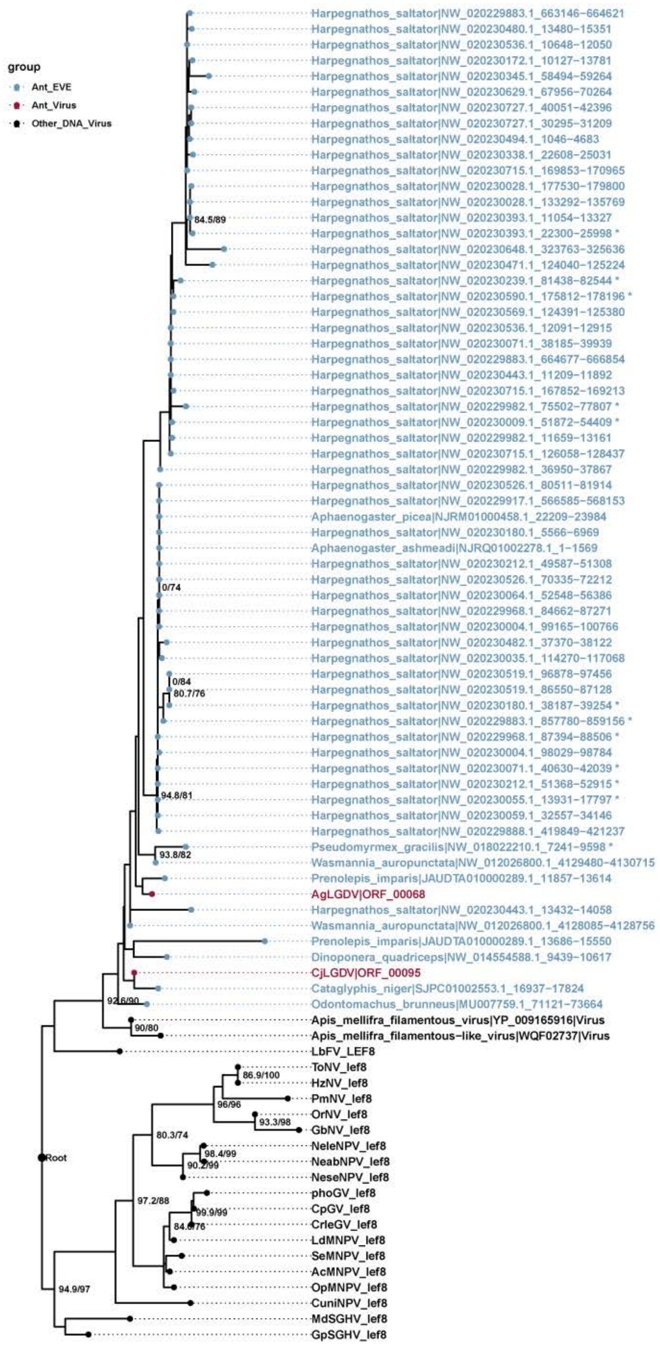

Figure S10-26 ATPase CjLGDV_ORF98

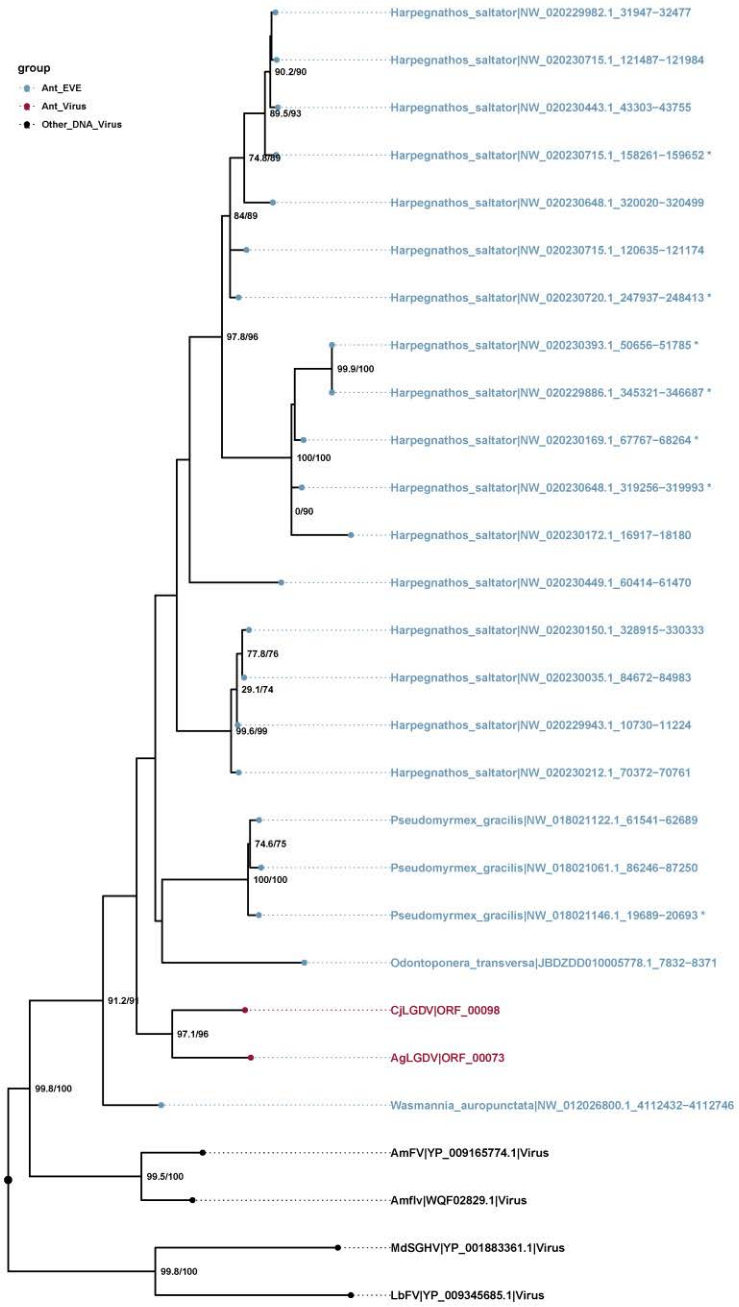

Figure S10-27 PIF1 CjLGDV_ORF105

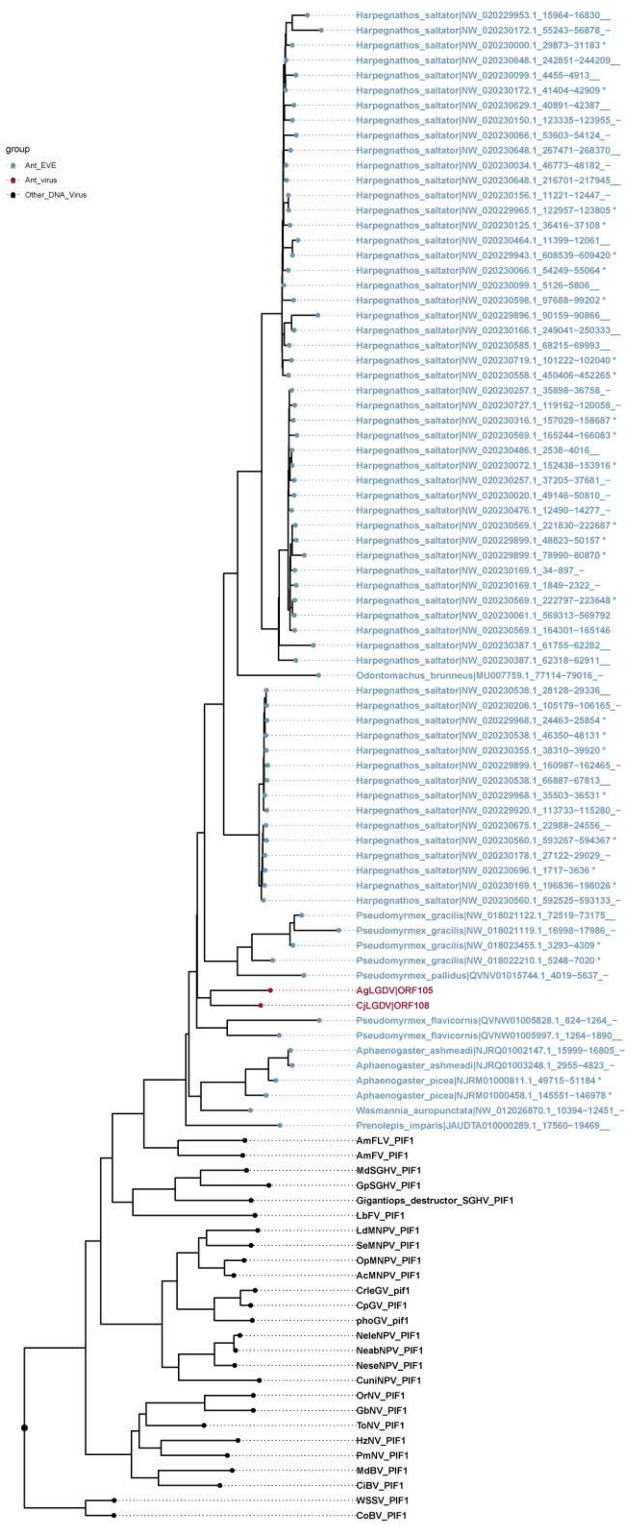

Figure S10-28 Ac81 CjLGDV_ORF110

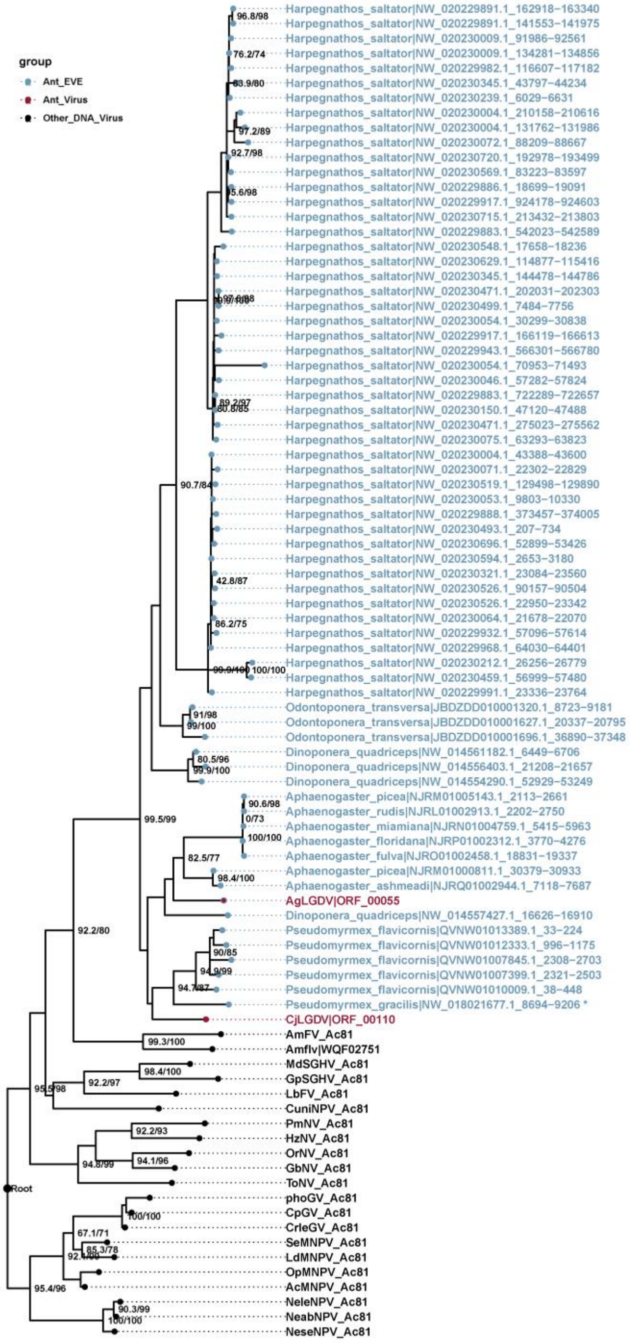

Supplementary Figure S10 Phylogenetic analysis of CjLGDV genes with EVEs detected in ant genomes. Different sources of sequences are indicated in different clolors: red for CjLGDV and AgLGDV, black for other viruses, and blue for ants. * denotes EVEs identified in a previous study. Support values are indicated as SH-aLRT support (%) / Ultrafast bootstrap (%).

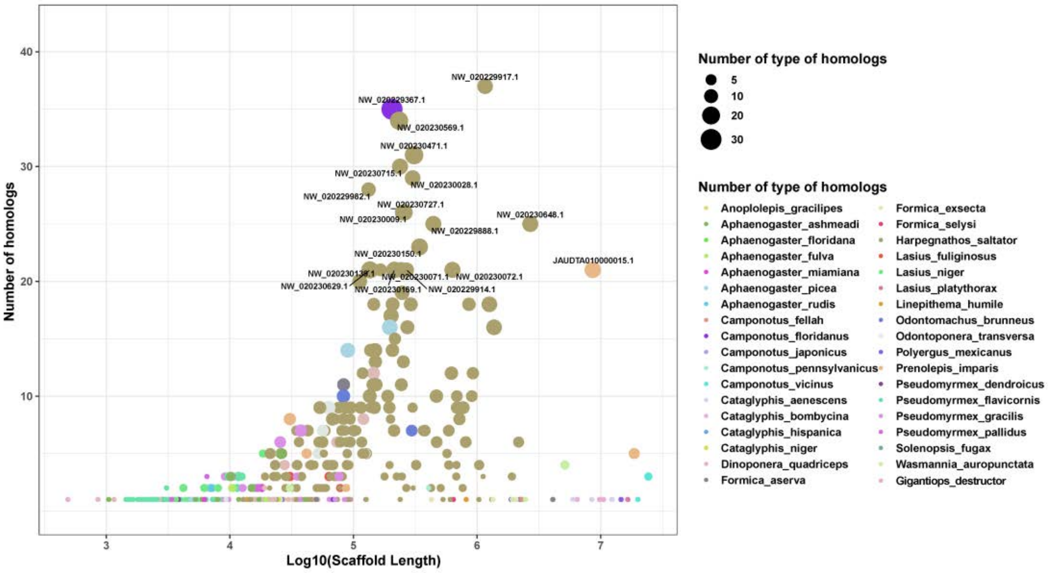

Supplementary Figure S11 Schematic for the relationship between scaffold length and the number of viral homologs in ant genomes. Dot colors represent different ant species, while dot sizes indicate the number of distinct types of homologs within the scaffolds.

A

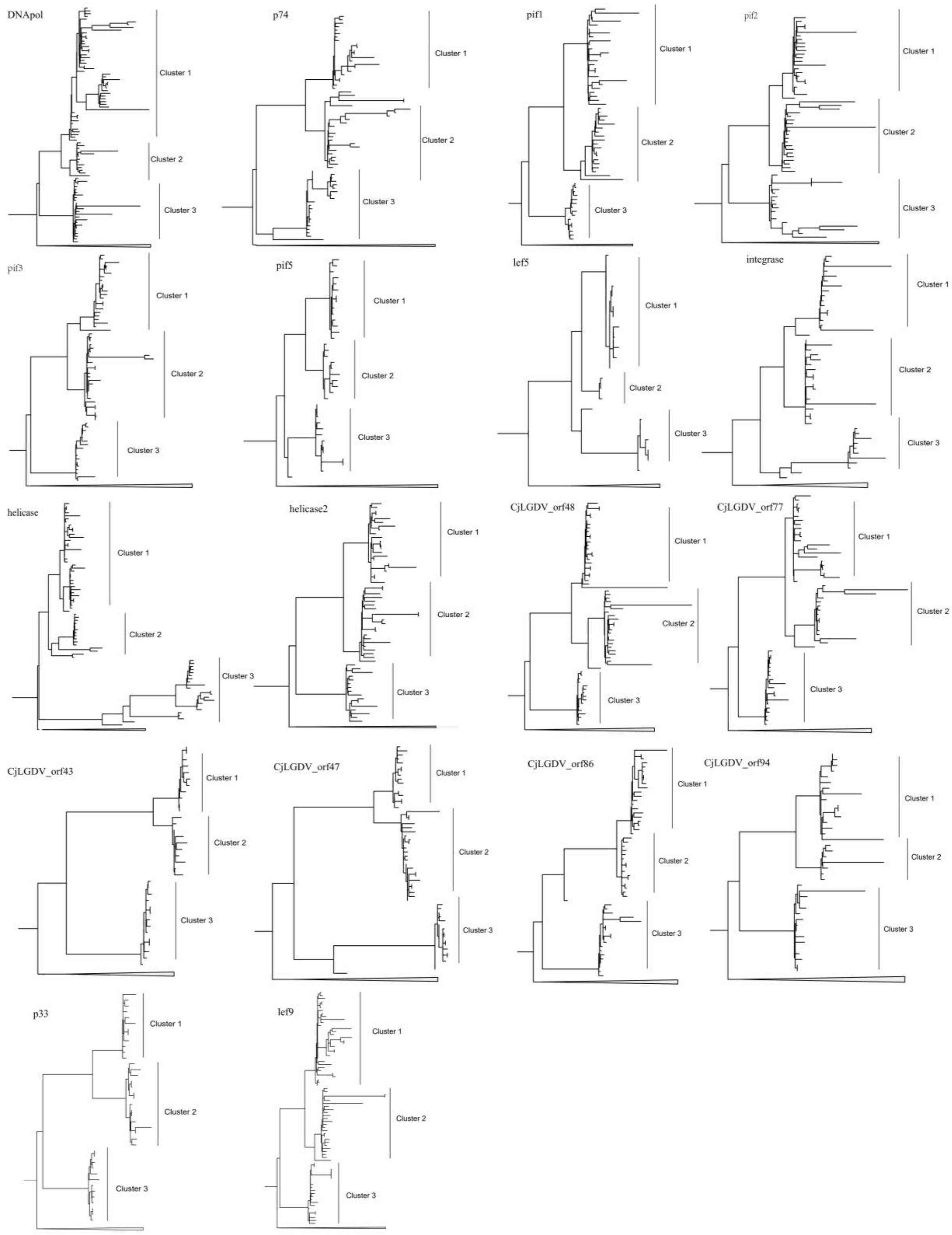

DNApol
Cluster 1
Cluster 2
Cluster 3
p74
Cluster 1
Cluster 2
Cluster 3
pif1
Cluster 1
Cluster 2
Cluster 3
pif2
Cluster 1
Cluster 2
Cluster 3
pif3
Cluster 1
Cluster 2
Cluster 3
pif5
Cluster 1
Cluster 2
Cluster 3
lef5
Cluster 1
Cluster 2
Cluster 3
integrase
Cluster 1
Cluster 2
Cluster 3
helicase
Cluster 1
Cluster 2
Cluster 3
helicase2
Cluster 1
Cluster 2
Cluster 3
CjLGDV_orf48
Cluster 1
Cluster 2
Cluster 3
CjLGDV_orf77
Cluster 1
Cluster 2
Cluster 3
CjLGDV_orf43
Cluster 1
Cluster 2
Cluster 3
CjLGDV_orf47
Cluster 1
Cluster 2
Cluster 3
CjLGDV_orf86
Cluster 1
Cluster 2
Cluster 3
CjLGDV_orf94
Cluster 1
Cluster 2
Cluster 3
p33
Cluster 1
Cluster 2
Cluster 3
lef9
Cluster 1
Cluster 2
Cluster 3

159

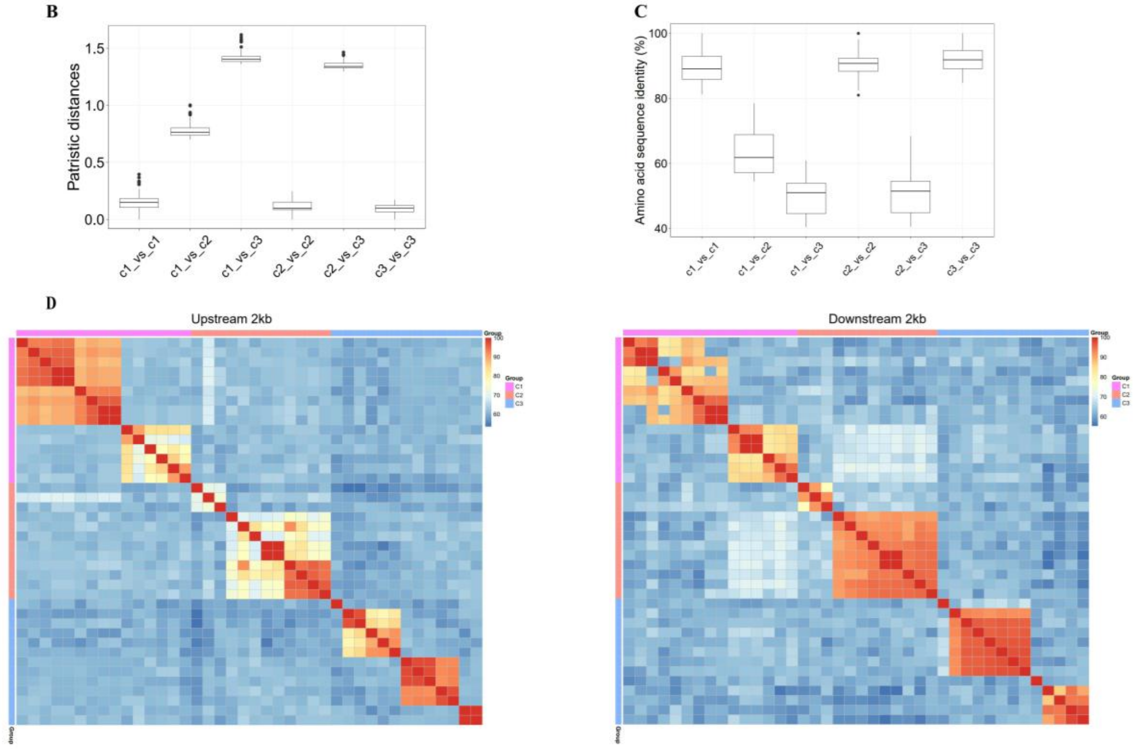

Supplementary Figure S12 Phylogenetic and sequence analyses of CjLGDV-related EVEs in the genome of *Harpegnathos saltator* suggest multiple endogenization events followed by gene duplication. (A) Maximum-likelihood phylogenetic tree of EVEs identified in *H. saltator*, showing clustering into three distinct groups, indicating at least three independent endogenization events. (B) Patristic distances within and between the three EVE clusters based on the *p33* homologs phylogenetic tree. (C) Amino acid sequence identity of *p33* homologs within and between clusters. (D) Heatmap of pairwise nucleotide identities of 2 kb genomic regions flanking *p33* EVEs.

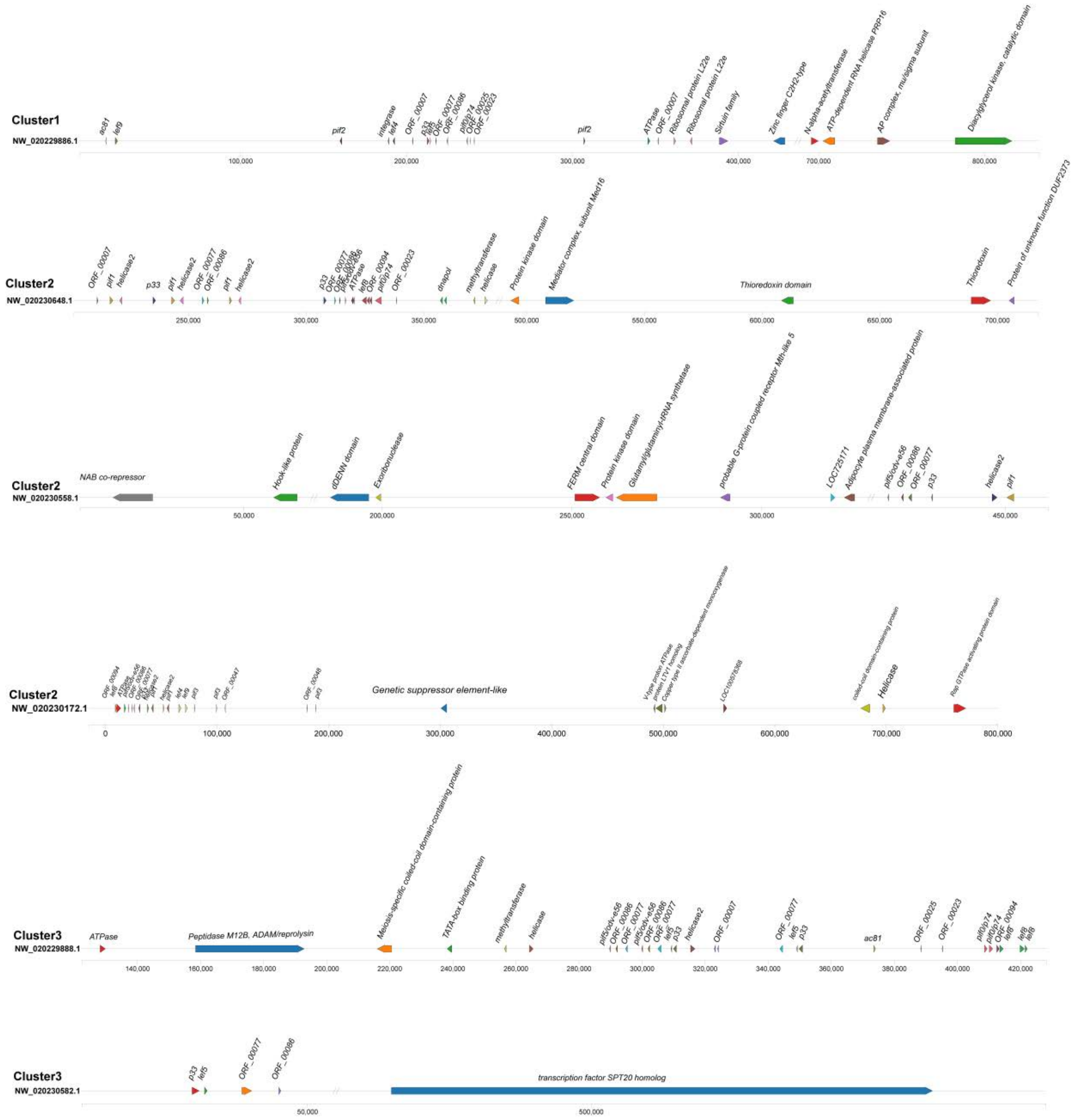

Supplementary Figure S13 Genomic environment for the endogenous viral elements (EVEs) detected in *H. saltator*. This figure shows the genomic scaffolds harboring viral *p33* homologs along with predicted eukaryotic genes. The EVE-containing scaffolds are grouped into Cluster 1, 2, or 3, as defined in Supplementary Figure S12A.

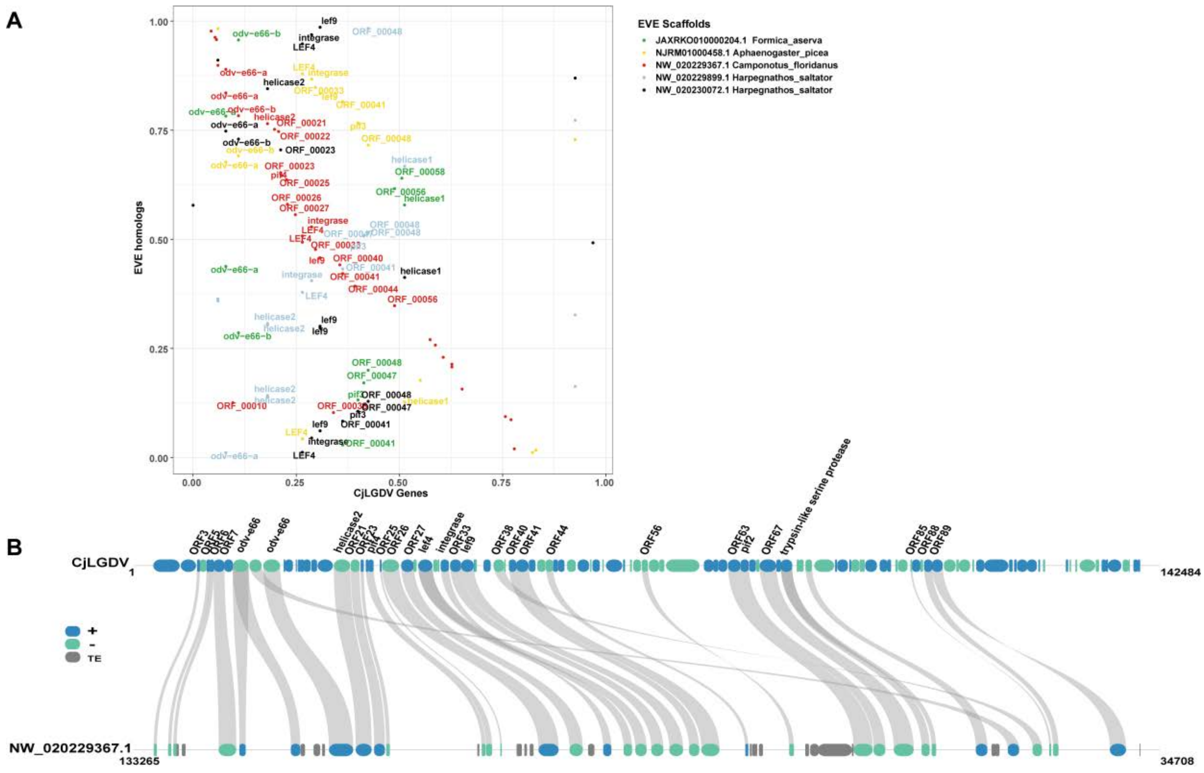

Supplementary Figure S14 Gene-order conservation between CjLGDV and EVEs. (A) Five representative EVEs showing conserved gene collinearity with CjLGDV. Each gene is represented by a dot, with the x-axis showing its order in the CjLGDV and the y-axis indicating the position of its homolog in EVEs. Different dot colors represent different EVEs. (B) A region in *Camponotus floridanus* genome showing high synteny with the CjLGDV genome. Homologs between the virus and ant are linked by gray lines. Blue and green ellipses represent genes on the positive and negative strand, respectively. Gray ellipses indicate transposable elements.

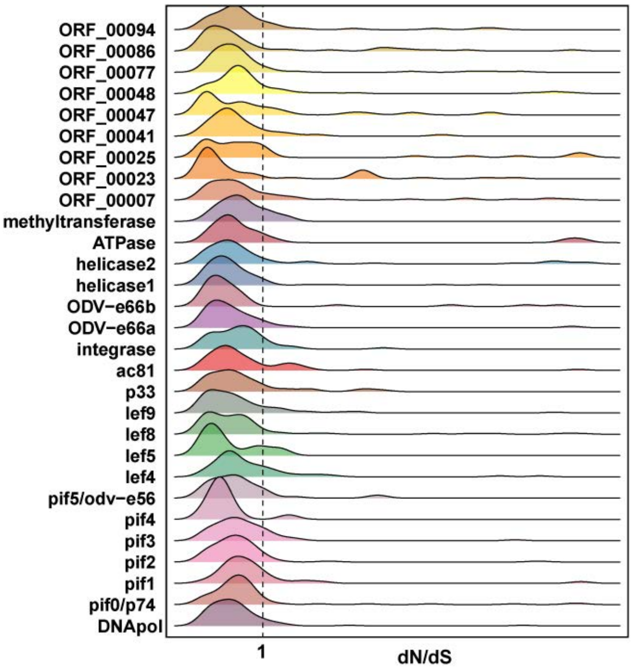

Supplementary Figure S15 The d_N/d_S analysis of viral homologs in EVEs. Density plot showing the distribution of d_N/d_S ratios derived from pairwise comparisons of homologs identified in EVEs across ant genomes. The X-axis represents the d_N/d_S ratio, a proxy for selective pressure: values <1 indicate purifying selection, ~1 suggest neutral evolution, and >1 imply potential positive selection.
